# Development of the self-directed RECEIVE Forgiveness workbook: a Christian-sensitive resource to support experiencing reconciliation with God

**DOI:** 10.3389/fpsyg.2025.1646103

**Published:** 2025-10-10

**Authors:** Richard G. Cowden, Jonathan C. Rutledge, Katherine Jackson-Meyer, Kaye V. Cook

**Affiliations:** ^1^Human Flourishing Program, Institute for Quantitative Social Science, Harvard University, Cambridge, MA, United States; ^2^Department of Epidemiology, Harvard T.H. Chan School of Public Health, Boston, MA, United States; ^3^School of Christian Studies, Asbury University, Wilmore, KY, United States; ^4^School of Psychology and Human Services, Gordon College, Wenham, MA, United States

**Keywords:** Christian flourishing, divine forgiveness, intervention, reconciliation with God, workbook

## Abstract

Divine forgiveness is a central concept in many theistic religions, including Christianity, which has a rich and longstanding history of doctrine, spiritual practices, and rituals addressing or reinforcing the idea that God forgives. Providing empirical support for the role that forgiveness from God plays in the lives of Christians, a growing body of literature suggests that divine forgiveness may have important implications for human flourishing. Practical resources that are biblically aligned and developed through interdisciplinary collaboration among humanists (e.g., theologians) and social scientists (e.g., psychologists) could complement traditional religious customs and equip healthcare professionals with spiritually integrated tools for serving self-identifying Christian clients. To this end, we developed the RECEIVE Forgiveness workbook as a brief, self-directed workbook intervention to support Christians in experiencing reconciliation with God after becoming aware of personal sin. This brief report describes the first phase of the workbook development process, including its theoretical foundations, central change objective, and engagement targets. After summarizing feedback from Christian religious leaders, healthcare professionals who serve self-identifying Christian clients/patients, and lay Christians, we describe how this input was incorporated into refinements aimed at strengthening the practical utility of the workbook. While rigorous empirical evaluation is needed to examine the efficacy of the RECEIVE Forgiveness workbook, we discuss the potential for this Christian-sensitive intervention to promote spiritual well-being and support flourishing among the Christian population.

## Introduction

Most of the global population identifies with a theistic religion that centers on belief in a supreme being or higher power that forgives (Cowden et al., 2025a). Christianity—the world’s largest religion—has a particularly robust theological framework for understanding God’s forgiveness (Cook and Cowden, 2025; Cowden et al., 2025b; Rutledge, 2022). A core aspect of the Christian faith is assurance of God’s forgiving character, a theme that is deeply woven into the biblical narrative and foundational to a Christian’s spiritual life (e.g., 1 John 1:9; Matthew 6:14–15). However, it is not uncommon for Christians to wrestle with a sense of dissonance between the doctrine of God’s forgiveness and their lived experience of it (Cook and Cowden, 2025; Hendricks et al., 2023). As one indirect indicator of this phenomenon, a recent ecological momentary assessment study of *n* = 104 United States Christian adults found that roughly one-third of personal transgressions meeting certain preconditions for divine forgiveness (e.g., confessing to God) were not accompanied by a subjective sense of having been forgiven by God (Currier et al., 2025). The gap between the ‘head’ and the ‘heart’ of divine forgiveness might be expressed in a variety of ways, such as: “I *know* that God forgives people for wrongdoing, but I just don’t *feel* as though God has forgiven me for what I’ve done wrong” (Cowden et al., 2025a). This dissonance has been conceptualized as a form of religious/spiritual struggle (i.e., tension, strain, or conflict around sacred matters), which has the potential to impact not only the spiritual dimension of a Christian’s life but also other key dimensions of their well-being too (Cook and Cowden, 2025; Pargament et al., in press). For example, a growing body of empirical research (mostly with Christian-majority samples) has shown that those who report lower divine forgiveness tend to endorse worse mental (e.g., more severe depression symptoms), social (e.g., greater loneliness), physical (e.g., more physical health problems), spiritual (e.g., greater religious/spiritual struggles), and overall well-being (e.g., Abernethy et al., 2022; Long et al., 2020; Tudder and Fincham, 2025). Such findings suggest that Christians (and people from other theistic faith traditions) might benefit from resources aimed at helping them bridge the gap between doctrine and experience through deeper internalization of and felt connection to God’s forgiveness. Toward this end, this brief report describes the development of the Christian-sensitive RECEIVE Forgiveness workbook.

### Relational spirituality and disruption to communion with God

The Christian life is grounded in relational spirituality, which emphasizes the believer’s personal relationship with God as the cornerstone of their spiritual journey. A central aim of the Christian spiritual journey is to experience communion with God, which might be defined as a mutual, intimate, and dynamic relationship initiated by God and reciprocated by the believer (Knabb and Wang, 2021). Cook and Cowden (2025) introduced the notion of a relational spirituality system to conceptualize the network of interconnected factors that can influence a Christian’s sense of communion with God. The system includes various elements across intrapersonal (e.g., doctrinal beliefs, religious identity), interpersonal (e.g., assumptions about human nature, relationships with other Christians), and transpersonal (e.g., perceptions of God’s character, spiritual experiences attributed to God) dimensions of a Christian’s life. These elements interact dynamically across space and time to form, maintain, and nurture a Christian’s relationship with God. The default state of the system is relative homeostasis—a somewhat stable internal condition characterized by harmony in a Christian’s relationship with God.

The stability of a Christian’s relational spirituality system can be supported through various mechanisms, such as prayer, reading the Bible and other related materials, corporate worship, or spending time in fellowship with other believers. However, there may be times when elements within the relational spirituality system disrupt a Christian’s relational harmony with God. One of the most common ways in which this occurs is through personal sin—when an individual becomes consciously aware that they have violated God’s values or standards for how people ought to live (McCall, 2019). Personal sin is often classified into sins of commission (i.e., to willfully think, say, or do something that goes against God’s values) and sins of omission (i.e., to fail to do something that God expects or commands), although not all personal sin can be neatly categorized. These transgressions may occur individually (e.g., harboring resentment) or collaboratively (e.g., conspiring with someone to deceive or harm another person).

### Experiencing reconciliation with God

When a Christian becomes consciously aware of personal sin that disrupts their relationship with God, stress-and-coping theory suggests that they may employ one or more coping strategies to deal with the distressing experience (Worthington et al., 2019). Integrating a relational spirituality lens with approach-avoidance theorizing on self-regulation, Christians might cope with such instances of personal sin in one of two broad ways: withdrawing from God (an avoidance orientation) or moving toward God (an approach orientation). For example, a Christian may choose to stop reading the Bible to avoid feelings of guilt (withdrawing from God), or they might confess their personal sin directly to God in an effort to begin repairing the relationship (moving toward God). The latter reflects the Christian notion of *engaging in repentance with God* (Cowden et al., 2025a), which might be broadly understood as turning back toward God after having turned away through personal sin (Heimann, 2022). Engaging in repentance with God is a multidimensional process—including cognitive, affective, and behavioral components—that can involve acknowledging the wrongdoing to God, experiencing remorse for having transgressed against God’s values, seeking help from God to make appropriate amends, and recommitting to live in accordance with God’s values (Cook and Cowden, 2025; Cowden et al., 2025a; Hendricks et al., 2023). Although this conceptualization of engaging in repentance with God is broadly ecumenical, there may be doctrinal variations in how Christians from different denominations experience these features and some Christian denominations may emphasize other features as well.

The process of restoring relational harmony with God after becoming consciously aware of personal sin also involves a parallel process in which a Christian perceives God moving toward them as they experience divine absolution for that personal sin. The concept of *experiencing absolution from God* for personal sin captures the process of change from a negative state of unexperienced forgiveness from God to a positive state of experienced forgiveness from God for that sin (Cowden et al., 2025a). It is a multidimensional process whose central features might include feeling cleansed by God from the guilt experienced because of personal sin, coming to experience peace with God over that sin, feeling freed by God from its burden, and experiencing a sense of assurance about God’s forgiveness for that sin (Cook and Cowden, 2025; Cowden et al., 2025a).

The culmination of both processes—engaging in repentance with God following recognition of personal sin and experiencing absolution from God—has been referred to as *experiencing reconciliation with God* (Cowden et al., 2025a). This experiential state is thought to strengthen the relative homeostasis of a Christian’s relational spirituality system through improved relational harmony with God (Cook and Cowden, 2025), which paves the way for deeper communion with God that can have positive downstream consequences for other dimensions of well-being. Although experiencing reconciliation with God is not the only possible outcome that may follow awareness of personal sin, it is generally considered a more adaptive outcome for bringing about renewed harmony to a Christian’s relationship with God and a heightened state of flourishing overall (Cowden et al., 2025a).

### A population health psychology approach to supporting reconciliation with God

The landscape of mental health treatment is undergoing considerable change as increasing calls are made for interventions that can overcome barriers to traditional treatment approaches and reach a broader population of individuals who may not seek treatment but might nonetheless benefit from supports (Dodge et al., 2024; Prochaska et al., 2020). For example, many people in need of mental health support are in the precontemplation phase of behavior change, choose not to seek treatment due to concerns about stigma, or find it challenging to access formal treatment due to financial constraints. There is also a growing emphasis on the need for spiritually integrated interventions that meaningfully engage with spiritually salient issues (e.g., religious/spiritual struggles) to support whole person functioning (Captari et al., 2022; Reist Gibbel et al., 2019). Although there are interventions available that attend to one of these issues, it is rare that interventions are both spiritually integrated and have the potential to be disseminated at scale. In this brief report, we describe the development of an intervention that addresses both of these needs.

Specifically, this brief report describes the development of the self-directed RECEIVE Forgiveness workbook—a freely available, low-intensity intervention to support Christians in experiencing reconciliation with God for personal sin they are struggling to feel absolved of. In developing this workbook intervention, we recognize there are many spiritual disciplines and rituals within the Christian faith that address or reinforce biblical teachings about God’s forgiveness. For example, the Sacrament of Holy Communion (i.e., taking of bread and wine to commemorate Jesus’ sacrifice) is a public ritual that is widely practiced on a regular basis across denominations to deepen the congregation’s sense of being forgiven by God. Other practices, such as the Sacrament of Confession (i.e., confession of personal sin to a priest who communicates absolution to the believer on behalf of God), tend to vary across denominations. Rather than replace these important and meaningful practices, the workbook intervention described in this brief report is intended to come alongside these practices to complement, expand upon, and enrich them through interdisciplinary engagement involving theology, philosophy, and psychology. In this way, the RECEIVE Forgiveness workbook might be considered a supplementary tool that Christian clergy may disseminate to parishioners in cases where it seems appropriate. The workbook intervention reported herein also offers mental health practitioners a tradition-specific resource that they could employ to support and engage with Christian clients on the topic of forgiveness from God, especially if the Christian faith does not fall within the practitioner’s spiritual competencies. The flexibility of the RECEIVE Forgiveness workbook means that it can be employed in various settings, ranging from an *ad hoc* resource in formal mental health treatment to a standalone self-help tool that Christians can work through independently. Since the workbook is freely accessible and adaptable for use in various formats (e.g., digital, web-based, printed out), it holds strong potential for widespread, low-cost dissemination.

In the sections that follow, we describe the process by which the RECEIVE Forgiveness workbook was developed, including its scope, theoretical underpinnings, central change objective, organization, and the engagement targets tied to each component of the workbook. We then summarize feedback that Christian laypeople, healthcare professionals who work with Christian clients/patients, and Christian religious leaders provided about the workbook, and discuss how we evaluated and used this feedback to refine and strengthen the workbook. We conclude with some discussion of the potential implications of this low-intensity intervention for restoring relational harmony with God and promoting flourishing in the Christian population.

## Workbook development process

Following the workbook intervention development template in Cowden et al. (2025c), we drew on the phases of the National Institutes of Health (NIH) Stage Model (Onken et al., 2014) to develop the RECEIVE Forgiveness workbook. This brief report addresses Stage I of the NIH Stage Model, including the design and refinement phase (Stage IA) supplemented with some initial feasibility and pilot testing (Stage IB). We document these activities and findings as a stepping-stone to future work focused on examining the efficacy of this intervention in research (Stage II) and community settings (Stage III).

The RECEIVE Forgiveness workbook was developed to serve as a low-intensity, self-directed intervention for Christian adults who may be struggling to fully experience absolution from God for a specific personal sin. Decisions about the scope of the workbook content and the mode of delivery were guided by our intention of creating a broadly ecumenical workbook that could be used widely by Christians from various denominational backgrounds. The two Christian theologians (second and third authors) involved in the development of the RECEIVE Forgiveness workbook possess expertise on the theology of divine forgiveness from training and engagement with core (and distinctive) doctrinal themes across a variety of Christian traditions, though we recognize that the workbook may not be fully exhaustive of nuanced doctrinal positions that may differ across some Christian denominations. We also do not intend for the workbook to be a replacement for spiritual care from Christian clergy or treatment from qualified healthcare professionals, although it may be a useful supplemental resource in many settings and instances. If the workbook is recommended to self-identifying Christians by individuals who interact with them as part of their vocation or profession, appropriate judgment should be used when making such decisions.

To strengthen the breadth of its applicability to the Christian population and accommodate differences in learning styles and preferences, we designed the RECEIVE Forgiveness workbook to engage individuals theologically, philosophically, and psychologically using various modalities (e.g., writing exercises, meditations, experiential activities). To enhance accessibility and flexibility, we chose to develop a digital version of the workbook for use with word processing programs (e.g., Microsoft Word) on computers, electronic tablets, or mobile devices, with the option to print for completion in hard copy format. This format also allows for adaptation to other modes of delivery, such as web-based platforms, which may further increase accessibility and provide opportunities to encourage engagement (e.g., automated reminders). Similar to other self-directed workbook interventions that have been developed previously, such as the REACH Forgiveness workbook (Ho et al., 2024) and the TRANSCEND Suffering workbook (Cowden et al., 2025c), the RECEIVE Forgiveness workbook was structured to be completed in a timeframe (i.e., 3–4 h) that provides an opportunity for individuals to experience meaningful change while also allowing them to incorporate their engagement with it into their daily lives.

### Theoretical underpinnings and central change objective

The RECEIVE Forgiveness workbook is grounded principally in the dual process model of experiencing reconciliation with God, a framework proposed to capture the key processes by which Christians might experience renewed harmony in their relationship with God following recognition of personal sin that introduces tension into their relational spirituality system (Cowden et al., 2025a). This framework was developed through interdisciplinary engagement with the intention of integrating Christian theology, philosophy, and psychology to provide a model that deals with the topic of divine forgiveness by attending to key priorities, needs, insights, and evidence reflected in each discipline. Following from this interdisciplinary conceptual foundation, the RECEIVE Forgiveness workbook brings together these disciplines to support a change process that is oriented toward the experience of reconciliation with God.

Drawing on Davis et al.’s (2021) monotheistic model of relational spirituality, our interdisciplinary approach to the workbook attempts to bring into closer alignment the doctrinal (i.e., how a Christian views God conceptually) and experiential (i.e., how a Christian relates with God) dimensions of a Christian’s relational spirituality system after relative homeostasis has been disrupted by recognition of personal sin. The doctrinal and experiential dimensions are complementary and interact dynamically (Knabb and Wang, 2021). The doctrinal dimension provides the intellectual scaffolding that shapes a Christian’s perspective of God and what it means to be a Christian, informing how they make sense of and explain what unfolds in the experiential dimension (e.g., “I felt a sense of peace when I prayed because I was reminded about scripture that says God has forgiven me through Christ”). However, the experiential dimension can also impinge upon the doctrinal dimension, such as when a Christian’s sense of guilt and shame challenges their ability to accept doctrine about God’s forgiveness for themselves (e.g., “I believe God forgives, but after what I’ve done I’m not sure that includes me”).

A relatively stable relational spirituality system suggests a high degree of successful integration of both the doctrinal and experiential dimensions. When a person becomes aware of personal sin, the potential disruption that this brings about to the relational spirituality system can lead to some degree of doctrinal-experiential disintegration (Cook and Cowden, 2025). The RECEIVE Forgiveness workbook was designed to strengthen doctrinal-experiential integration by bringing together Christian theology, philosophy, and psychology in ways that engage the doctrinal and/or experiential dimensions at different points during the process of completing it. For example, the workbook engages the doctrinal dimension by using both theology and philosophy to clarify or reinforce how divine forgiveness is understood within the Christian faith; it also engages the experiential dimension by using insights from psychology to provide individuals with the opportunity to relate to God in new or different ways. By engaging both the doctrinal and experiential dimensions, the workbook has the potential to support doctrinal-experiential reintegration among a diverse range of individuals who begin the workbook at different places of doctrinal-experiential disintegration. Against this backdrop, the central change objective of the RECEIVE Forgiveness workbook is to support individuals who identify as Christian in experiencing reconciliation with God by repairing doctrinal-experiential disintegration in their relational spirituality system that was introduced after becoming aware of personal sin.

### Workbook structure and engagement targets

We organized the RECEIVE Forgiveness workbook into seven components that align with its central change objective: **R**emember God’s love for you; **E**xplore how God forgives; **C**onfront your sin; **E**ngage with God to make amends; **I**nternalize God’s forgiveness; **V**enture toward renewal and virtue; and **E**stablish a plan to keep experiencing God’s forgiveness. Each component is structured around two engagement targets that guide the scope, structure, and activities used to facilitate change (see Table 1). The workbook was developed by a team with expertise in psychology, Christian theology, and philosophy who contributed their theoretical, empirical, and/or clinical expertise through a collaborative engagement process. The engagement targets guided decisions about the content and activities that form the key ingredients for each component, but some exercises may intersect multiple targets rather than aligning strictly with one. The workbook engages a variety of modalities—ranging from written assignments to meditations to experiential activities—to promote change in alignment with the engagement targets for each component.

**Table 1:** RECEIVE Forgiveness workbook components, engagement targets, and scriptural anchoring.

Component	Engagement target	Scriptural anchoring
**R**emember God’s love for you	Deepens their understanding of God’s loving character. Experiences a stronger personal connection to God’s love.	John 3:16–17 Luke 15:11–32 Romans 8
**E**xplore how God forgives	Understands more fully that their personal struggles with transgressing God’s values is a shared human experience and all people are in need of forgiveness from God. Becomes more consciously aware that it is in God’s loving nature to forgive and that God’s forgiveness is available.	Acts 9:1–19 Psalm 86:5 Psalm 103:8–12
**C**onfront your sin	Takes appropriate responsibility for a specific transgression they are struggling with. Gains further insight into the personal, social, and spiritual impacts of the transgression to uncover opportunities for personal or spiritual growth.	Psalm 51 Luke 23:39–43 Proverbs 28:13
**E**ngage with God to make amends	Identifies personal and communal avenues they could pursue with God to make amends for the transgression. Pursues a God-centered approach to try to make amends for the transgression.	Luke 19:1–10 2 Corinthians 5:17–19 Matthew 5:23–24
**I**nternalize God’s forgiveness	Experiences a reduction in negative thoughts and/or feelings over the transgression. Experiences a positive change in their internal acceptance of God’s forgiveness for the transgression.	Mark 1:40–45 Galatians 2:20 2 Timothy 1:12 Luke 7:47
**V**enture toward renewal and virtue	Identifies and pursues personal and/or communal activities that support the development of virtuous habits. Makes a (renewed) commitment to live in a way that reflects God’s values.	Matthew 26:39 Philippians 4:8–9 2 Peter 1:5–8
**E**stablish a plan to keep experiencing God’s forgiveness	Identifies and plans for the use of suitable strategies to address future doubts about whether God has forgiven them for the transgression. Strengthens their connection to positive experiences and changes arising from the process of engaging with the workbook.	Luke 24:1–33 Hebrews 10:23–25 Romans 8:31–39

Although the RECEIVE Forgiveness workbook is a novel intervention grounded principally in relational spirituality (Davis et al., 2021) and the dual process model of experiencing reconciliation with God (Cowden et al., 2025a), its components are compatible with well-established theoretical and therapeutic models. The literature on religious/spiritual struggles (e.g., explore how God forgives), positive religious coping (e.g., engage with God to make amends), meaning-making (e.g., internalize God’s forgiveness), and virtue ethics (e.g., venture toward renewal and virtue) offer some examples of the extended theoretical linkages to components of the workbook (Pargament, 1997; Pargament and Exline, 2022; Park, 2010; Peterson and Seligman, 2004). Psychotherapeutically, components of the RECEIVE Forgiveness workbook intersect with mindfulness-based stress reduction (e.g., remember God’s love for you), cognitive behavior therapy (e.g., confront your sin), emotion-focused therapy (e.g., internalize God’s forgiveness), and acceptance and commitment therapy (e.g., venture toward renewal and virtue) approaches that are commonly applied by psychological professionals (Beck, 2021; Greenberg, 2017; Hayes, 2005; Kabat-Zinn, 2005). These examples of connecting theory and therapeutic models are intended to be representative rather than exhaustive, providing an illustrative sampling of how the workbook ties into the wider landscape of psychological science.

### Workbook feedback and refinement

After the interdisciplinary group of authors constructed an initial version of the RECEIVE Forgiveness workbook, feedback was sought from Christian religious leaders and healthcare professionals who are familiar with the Christian faith and provide services to individuals identifying as Christian. This feedback was solicited as part of an initial social validity process undertaken to assess the acceptability, relevance, and perceived usefulness of the workbook among relevant stakeholders and the target population of users (Kazdin, 1977). Using our personal and professional networks, we identified *n* = 5 Christian religious leaders (i.e., two doctoral-level pastor-theologians, two pastors, and one pastor-chaplain)—representing a combination of evangelical Protestant (e.g., Christian Missionary Alliance, Evangelical Covenant Church) and more historically rooted or liturgical traditions (e.g., Anglican, Catholic)—and *n* = 7 healthcare professionals (i.e., four psychologists, two physicians, and one psychiatrist) in the United States who were willing to evaluate and provide feedback on the workbook. We also leveraged contacts in our extended social networks to disseminate the workbook to Christians and invite feedback from those interested in completing the RECEIVE Forgiveness workbook. Anonymous feedback was received from *n* = 23 United States adult Christians with diverse denominational affiliations (e.g., Anglican, Baptist, Presbyterian, Eastern Orthodox).

Religious leaders and professionals were asked to respond to several Likert-type items intended to gauge whether they considered the workbook a potentially suitable resource for typical parishioners or Christian clients/patients with whom they work (e.g., “The workbook is an appropriate resource for [people in my congregation]/[clients/patients I work with who identify as Christian]”), which they rated using a five-point response scale (1 = *strongly disagree*, 5 = *strongly agree*). Each item was followed by a prompt requesting further explanation for their choice. Some religious leaders/professionals provided additional feedback through comments appended to the workbook itself, which were also considered. The lay Christians who agreed to provide feedback were given a set of guiding questions (with follow-up prompts requesting further explanation) that asked them to reflect on their experience of going through the workbook (e.g., “Did you experience any challenges completing the exercises in the workbook? If so, which one/s were challenging?”), along with several related Likert-type items (e.g., “The workbook helped me to connect more deeply with God’s forgiveness”) that they rated using a five-point response scale (1 = *strongly disagree*, 5 = *strongly agree*). While the feedback from these two groups was an important part of refining the RECEIVE Forgiveness workbook, the relatively small sample sizes and convenience-based recruitment approach should be taken into account when interpreting the feedback we received.

Summary statistics for the rating scale items, presented separately for Christian religious leaders/healthcare professionals and lay Christians involved in this initial piloting process, are reported in Table 2. Religious leaders/professionals generally found the workbook to be a suitable resource for the Christian population and indicated a willingness to give it to Christians they engage with as part of their vocation or profession (means ranging from 4.55 to 4.73 across the items). Lay Christians indicated that the workbook is relevant to their lives, their experience completing it helped them to internalize God’s forgiveness for a specific personal sin they have been struggling with, and they would recommend it to other Christians (means ranging from 4.26 to 4.61 across the items).

**Table 2:** Summary statistics for rating scale feedback.

Item	*M* ± *SD* (observed range)
Christian religious leaders and healthcare professionals	
The workbook is an appropriate resource for [people in my congregation]/[clients/patients I work with who identify as Christian].	4.55 ± 0.69 (3–5)
The workbook would be helpful to [people in my congregation]/[clients/patients I work with who identify as Christian].	4.73 ± 0.47 (4–5)
I would be willing to give the workbook to [people in my congregation]/[clients/patients I work with who identify as Christian].	4.55 ± 0.69 (3–5)
I would recommend the workbook to other [Christian religious leaders]/[practitioners in my field who provide services to self-identifying Christians].	4.73 ± 0.47 (4–5)
Lay Christians	
The workbook addresses a topic that is relevant to my life.	4.30 ± 0.76 (3–5)
The workbook is consistent with my cultural beliefs and values.	4.61 ± 0.66 (3–5)
I liked the exercises used in the workbook.	4.26 ± 0.69 (3–5)
The workbook helped me to connect more deeply with God’s forgiveness.	4.41 ± 0.73 (3–5)
I would recommend the workbook to other Christians.	4.57 ± 0.59 (3–5)

*M*, mean; *SD*, standard deviation. Response options for each item range from 1 (*strongly disagree*) to 5 (*strongly agree*).

We synthesized responses to the open-ended questions using thematic analysis to identify key themes (Braun and Clarke, 2006). Given the differences in questions used to solicit feedback from religious leaders/professionals compared to lay Christians, the feedback received from these groups was synthesized separately. The first three authors engaged independently with the feedback from each group to gain familiarity with it. After meeting together to discuss the feedback, they adopted an approach that prioritized themes consistent with the central change objective and engagement targets of the workbook. Any disagreements were resolved through consensus. Themes that emerged for religious leaders/professionals included (1) theological emphases (e.g., clarify concepts that could lead to theological misinterpretations or imbalance, make the Crucifixion more central); (2) practical considerations (e.g., the self-directed format may have limits without emotional or spiritual safety from clergy, alternative language choices may strengthen how people connect with the resource); and (3) suggestions for dissemination (e.g., potential for the workbook to be used in small groups within a church, possibility for increased engagement by reducing or condensing the workbook). Themes that emerged based on the feedback received from lay Christians included (1) challenges completing the workbook (e.g., complex language introduced confusion at times, some exercises had lengthy instructions, overall length); (2) suggestions for improving engagement (e.g., reduce repetitive content, modify language to make the workbook more personal, consider making the design and layout more appealing); and (3) strengths of the workbook (e.g., multiple mediums/modalities, positive emotional or spiritual impact, engaging material). Elements of the workbook that introduced ideas that challenged users (e.g., Lesson 2), prompted deeper reflection on or engagement with a specific personal sin (e.g., Lesson 5), and required more consistent daily engagement (e.g., Lesson 10) were noted by some users as being more difficult to complete.

All feedback was reviewed and integrated into the revised version of the RECEIVE Forgiveness workbook, except in rare cases where there was collective agreement that integrating certain feedback might detract from the workbook’s central change objective or engagement targets (e.g., suggestions to remove content that was considered theologically essential). The team involved in developing the workbook engaged in a collaborative process to determine appropriate refinements aimed at improving the clarity, accessibility, and potential effectiveness of the workbook. The refinements can be organized thematically into (1) clarifying and simplifying language; (2) making adjustments to some of the exercises; and (3) including additional examples and statements of encouragement in strategic places. Table 3 summarizes the type and extent of these refinements, along with selected examples of each. The final Microsoft Word-compatible (English) version of the RECEIVE Forgiveness workbook can be freely accessed and downloaded via https://osf.io/dv85a/ (see also Supplementary material).

**Table 3:** Summary of revisions to the RECEIVE Forgiveness workbook.

Revision type	Component affected (section/lesson revised)	Representative examples of revisions
Clarification and simplification of language	Prelude (Setting the stage) Explore how God forgives (Lesson 3) Confront your sin (Lesson 5) Venture toward renewal and virtue (Lesson 10)	Simplified the RECEIVE acrostic (Setting the Stage). Adjusted framing of the David and Bathsheba story to avoid contextual misinterpretation (Lesson 3, Exercise 3.2). Simplified the theological discussion of Reinhold Niebuhr and focused on personal sin as a form of pride or idolatry (Lesson 5, Exercise 5.2). Changed language from ‘covenant’ to ‘commitment’ to avoid potentially distracting theological debates (Lesson 10, Exercise 10.6).
Adjustments to exercises	Explore how God forgives (Lesson 4) Confront your sin (Lesson 5) Confront your sin (Lesson 6) Engage with God to make amends (Lesson 7) Internalize God’s forgiveness (Lesson 8) Internalize God’s forgiveness (Lesson 9) Venture toward renewal and virtue (Lesson 10)	Expanded advice about discerning whether or not to share the apology with the person(s) affected by the personal sin (Lesson 7, Exercise 7.5). Created an audio meditation option (Lesson 8, Exercise 8.1). Removed some recollection from childhood material and focused on the qualities Christ sees in them (Lesson 9, Exercise 9.3). Drew a stronger connection between the Examen practice and experiencing divine forgiveness (Lesson 10, Exercises 10.2–10.4).
Addition of examples and encouragement statements	Confront your sin (Lesson 5) Internalize God’s forgiveness (Lesson 8) Internalize God’s forgiveness (Lesson 9)	Added examples from Augustine of Hippo, Timothy Keller, and John Calvin to provide different ways of thinking about personal sin (Lesson 5, Introduction). Reiterated holding onto God’s love while working through the chosen personal sin (Lesson 5, Exercise 5.1). Added a paragraph to encourage participants as they work through their chosen personal sin (Lesson 8, Introduction). Included a closing prayer with reflections from scripture (Lesson 9, Exercise 9.5).

## Discussion

The RECEIVE Forgiveness workbook is a novel intervention that was developed to bridge the gap between calls for a population health psychology approach to interventions and the need for spiritually integrated interventions that engage more deeply with salient spiritual issues to promote wellbeing. As an interdisciplinary intervention that brings together Christian theology, philosophy, and psychology, this freely available low-intensity intervention is a biblically aligned resource to support Christians in their experience of reconciliation with God following recognition of personal sin. This brief report documented the initial phase of intervention developed that was undertaken as part of Stage I of the NIH Stage Model, laying the foundation for subsequent work (i.e., Stage II and/or III) focused on evaluating the efficacy of this promising intervention.

### Anticipated proximal and distal effects

With the conceptual underpinnings of the RECEIVE Forgiveness workbook grounded in the biblically aligned dual process model of experiencing reconciliation with God following recognition of personal sin, the workbook is expected to produce some combination of positive change in the two central dimensions of the model—engaging in repentance with God and experiencing absolution from God. Improvements along these lines may be somewhat heterogeneous depending on the state of a Christian’s relational spirituality system at the time they begin to engage with the workbook. For example, if people have already engaged in some degree of repentance with God but have yet to fully experience absolution from God, their engagement with the workbook may principally bring about deeper internalization of God’s forgiveness. On the other hand, both engaging in repentance with God and experiencing absolution from God may show meaningful change when each is relatively absent prior to beginning the workbook.

Even if entry points to the RECEIVE Forgiveness workbook vary, prior theorizing suggests that improvement in the homeostasis of a person’s relational spirituality system brought about by experiencing reconciliation with God for personal sin sets the stage for renewed or deeper communion with God (Cook and Cowden, 2025). Thus, we might reasonably anticipate that experiencing reconciliation with God would precipitate improvements in subjective evaluations of communion with God. The workbook is also likely to have other proximal effects, particularly on factors more closely connected to the relational spirituality system. For example, people may come to view God as more benevolent, uncover a deeper layer of awe or gratitude toward God, or experience a general decline in the religious/spiritual struggles they were encountering before completing the workbook. Some of these changes may also function as mechanisms by which the RECEIVE Forgiveness workbook facilitates experiencing reconciliation with God. To illustrate, a shift toward a more benevolent view of God might open some people up to more fully internalizing God’s forgiveness for personal sin. As with most interventions, a combination of mechanisms is likely to be operative. Specifying engagement targets and aligning each component of the workbook with those targets provides an opportunity to explore which mechanisms of change are operative and how they might vary across different components of the workbook.

Although the RECEIVE Forgiveness workbook is likely to have the most proximal and direct effects on the spiritual dimension of functioning, those effects may trickle down into positive changes to more distal dimensions of well-being. For instance, an increase in the stability of the relational spirituality system brought about by experiencing reconciliation with God might lead to better mental health and psychological well-being (Abernethy et al., 2022; Fincham and May, 2024). Social aspects of well-being might also improve, not only through the possibility of restoring relationships that have been strained by personal sin but also through transformed patterns of relating to others that often follow profound experiences of reconciliation with God (Hendricks et al., 2023; Long et al., 2020). To the extent that the tension introduced into the relational spirituality system following recognition of personal sin is acute and has lingered in an unresolved state, the impacts of experiencing reconciliation with God may accrue benefits to some aspects of physical well-being (Bassett et al., 2016, Study 2; Upenieks, 2021). While we are not claiming that the RECEIVE Forgiveness workbook will directly address every aspect of whole person functioning, the experience of reconciliation with God that it intends to facilitate may serve as a catalyst for broader positive changes in personal flourishing.

### Flexibility and scaling possibilities

**Figure 1:**
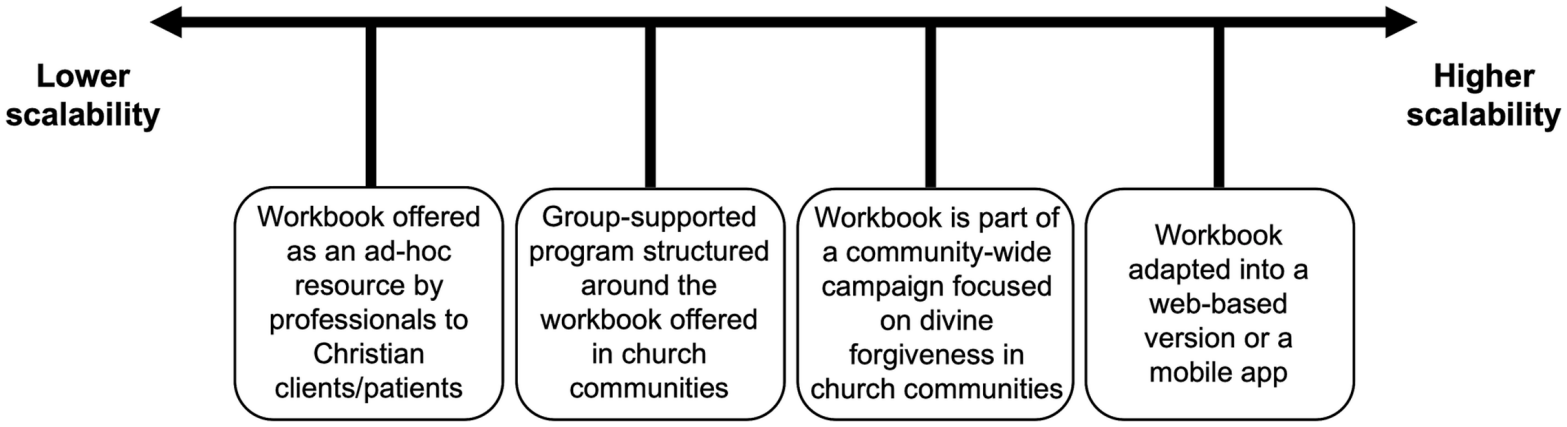
RECEIVE Forgiveness workbook dissemination possibilities.

As a brief, self-directed intervention, the dissemination potential of the RECEIVE Forgiveness workbook is high and could be employed flexibly at varying degrees of scale. Figure 1 offers some selective examples to illustrate the flexibility of the workbook and possibilities for dissemination. At a more individual level (lower scalability), the workbook could be offered by healthcare professionals as a resource to Christian clients/patients with whom they work. This may require some insight into whether the client/patient is struggling spiritually because of personal wrongdoing and is interested in exploring the workbook as a potential resource. A moderately scalable option is to use the workbook as the foundation for a group-supported program in a church setting where a small group of parishioners completes the workbook independently and periodically gather together to reflect, share experiences, and encourage one another under the guidance of a facilitator or peer leader. To expand its reach further, the RECEIVE Forgiveness workbook could serve as part of a community-wide campaign where the entire congregation spends a designated period of time focusing on the topic of divine forgiveness in different ways. Although the workbook is an individual intervention, the benefits to the individual might translate into a more flourishing church community when it is completed as part of a broader collective within the context of a supportive church group or entire congregation. At a higher level of scalability, the workbook could be adapted into a freely accessible web-based platform or mobile app that individuals could access and return to at their convenience. A web-based version of the RECEIVE Forgiveness workbook is currently in development, which may offer the greatest global accessibility if it can be made available in multiple languages.

Although the flexibility and scalability of low-intensity self-directed interventions like the RECEIVE Forgiveness workbook are important strengths, these features alone may not be sufficient for achieving high uptake. To strengthen the utility of this workbook as a resource for Christian populations, dissemination and implementation strategies should bridge the gap between an intervention’s availability and its actual use. A multipronged approach, such as offering the workbook through a web-based platform, via group-supported programs in church communities, and community-wide campaigns within congregations, may help to scale it in a way that increases the likelihood of sustained engagement to completion.

### Reflection on interdisciplinarity in developing psychospiritual interventions

A topic as theologically rich and existentially significant as divine forgiveness calls for careful attention to the theological foundations and insights that shape how individuals within a specific faith tradition understand and relate to the sacred. For Christians, beliefs about divine forgiveness are deeply embedded in doctrinal teachings, sacred texts, and communal practices, all of which influence how a person understands, responds to, and transcends personal sin through their relationship with God. The RECEIVE Forgiveness workbook represents one of the first intentional efforts to integrate theological/philosophical reflection and psychological science in a tradition-specific intervention aimed at supporting reconciliation with God for personal sin. While further empirical work is needed to evaluate the efficacy of this low-intensity, self-directed intervention among Christians, the workbook development process undertaken in this brief report illustrates the value of an interdisciplinary approach when addressing spiritual concerns that are central to a faith tradition and can have powerful impacts on people. Neglecting the theological dimension in interventions targeting concepts like divine forgiveness risks diminishing their relevance and importance for those whose spiritual lives are shaped by longstanding religious traditions. Underemphasizing or overlooking theology may also reduce the capacity of an intervention to facilitate meaningful change among the target population it is intended to serve. We hope that the process by which the RECEIVE Forgiveness workbook was developed may encourage other interventionists to draw on the wisdom of both the humanities and the social sciences when approaching topics like divine forgiveness.

### Limitations and caveats to consider

The RECEIVE Forgiveness workbook was developed as a Christian-sensitive resource. While we believe that this is a key strength of the workbook, one important trade-off of developing a tradition-specific intervention is that its suitability for those who identify with other faith traditions or religiously diverse populations is more limited. Based on prior research pointing to potential benefits of engaging in repentance and experiencing divine forgiveness in the context of other theistic religions, such as Islam (e.g., Uyun et al., 2019), adaptations of the RECEIVE Forgiveness workbook could be considered for non-Christians who believe in a higher power or supreme being that forgives.

Although the RECEIVE Forgiveness workbook was designed with the intention of having broad utility in the Christian population, it may not be appropriate for all individuals who are struggling to fully experience absolution from God for personal sin. For example, it may not be a suitable resource for Christians struggling with addiction or clinically severe scrupulosity because they may need more formal, structured support from qualified healthcare professionals. While we have proposed that the RECEIVE Forgiveness workbook may serve as a potentially useful resource that could be offered by Christian religious leaders and healthcare professionals to self-identifying Christians they interact with as part of their vocation or profession, careful judgment must be applied when making decisions about whether to recommend the workbook to someone and how best to support them as they engage with it. When the RECEIVE Forgiveness workbook is recommended, it should be presented as a supportive tool rather than a prescriptive formula, recognizing that the process of experiencing forgiveness from God is shaped by each person’s unique and deeply personal relationship with God.

The RECEIVE Forgiveness workbook was developed by a group of theologians, philosophers, and psychologists from the West. Although the theology that underpins the workbook is not unique to Christianity in the Western context, Western-centric influences are likely to have shaped the workbook and its content. For example, some content or activities in the workbook may have Western cultural references that might not be widely known among people living outside the Western context, suggesting that some caution may be needed when using the workbook among Christians living in non-Western contexts. Cultural adaptation or modification of the RECEIVE Forgiveness workbook might be an interesting and important direction for future work. While the workbook was developed to be broadly ecumenical, it may not be possible for a brief workbook intervention to satisfy all Christian denominational nuances or variations pertaining to doctrine on forgiveness from God.

Although it is not uncommon for Christians to struggle with experiencing absolution from God for personal sin, experiences can be quite different across individuals (Cowden et al., 2025a). It is reasonable to expect some heterogeneity in the way that individuals resonate with or benefit from the RECEIVE Forgiveness workbook, particularly if their entry point to the workbook differs. For instance, one person may experience reconciliation with God through exposure to theological material in the workbook that strengthens or expands their understanding of God’s forgiveness (i.e., the doctrinal dimension), whereas another might come to experience reconciliation with God through workbook exercises that help them connect God’s forgiveness more directly to their own life (i.e., the experiential dimension). Rigorous research is needed to determine how different factors (e.g., individual, developmental, denominational, cultural) might contribute to variability in the effects of the RECEIVE Forgiveness workbook, which could be helpful in identifying segments of the Christian population that may be especially likely to benefit from the workbook (or need more tailored interventions). It would also be useful to explore how different approaches to implementing the workbook (e.g., completing it individually versus within a group-supported setting) might affect outcomes, as those insights could be valuable for enhancing its practical utility. Incorporating longer-term follow-up into intervention studies examining the efficacy of the workbook will be important for evaluating the durability of its potential benefits over time.

The self-directed nature of the RECEIVE Forgiveness workbook offers practical advantages, such as ease of access and low cost of making it available, but a key limitation of self-guided interventions is the lack of individualized feedback and support that can help maintain engagement and encourage progress (Mohr et al., 2011). Without guidance or encouragement, some individuals may lose motivation or struggle to complete the intervention (Donkin et al., 2011). These common limitations associated with self-directed interventions may also be relevant when considering the potential challenges people might face while completing the RECEIVE Forgiveness workbook. Future work could consider monitoring how users engage with the workbook and identifying opportunities to encourage and sustain engagement.

## Conclusion

In this brief report, we described the initial phase of developing the RECEIVE Forgiveness workbook, a freely available self-directed intervention that seeks to provide those who identify with the Christian tradition an opportunity to experience reconciliation with God following personal sin. Although research employing rigorous methodologies (e.g., randomized controlled trials) is needed to evaluate the efficacy of the RECEIVE Forgiveness workbook, this novel low-intensity intervention shows promise as a flexible and scalable resource. By developing an interdisciplinary resource that brings together psychological, theological, and philosophical perspectives on divine forgiveness, the RECEIVE Forgiveness workbook adds to a growing body of spiritually integrated interventions seeking to promote human flourishing.

## Data Availability

The original contributions presented are included in the article or Supplementary material. Further inquiries can be directed to the corresponding author.
