# Supplementary material for: Development of the self-directed RECEIVE Forgiveness workbook: a Christian-sensitive resource to support experiencing reconciliation with God
Source: Front Psychol. 2025 Oct 10;16:1646103. doi: 10.3389/fpsyg.2025.1646103 (PMC12549654; doi:10.3389/fpsyg.2025.1646103)
Supplement: Supplementary file 1 [file Table_1.DOCX]

**RECEIVE Forgiveness**

Self-Directed Exercises to Guide Your Journey of Experiencing God’s Forgiveness


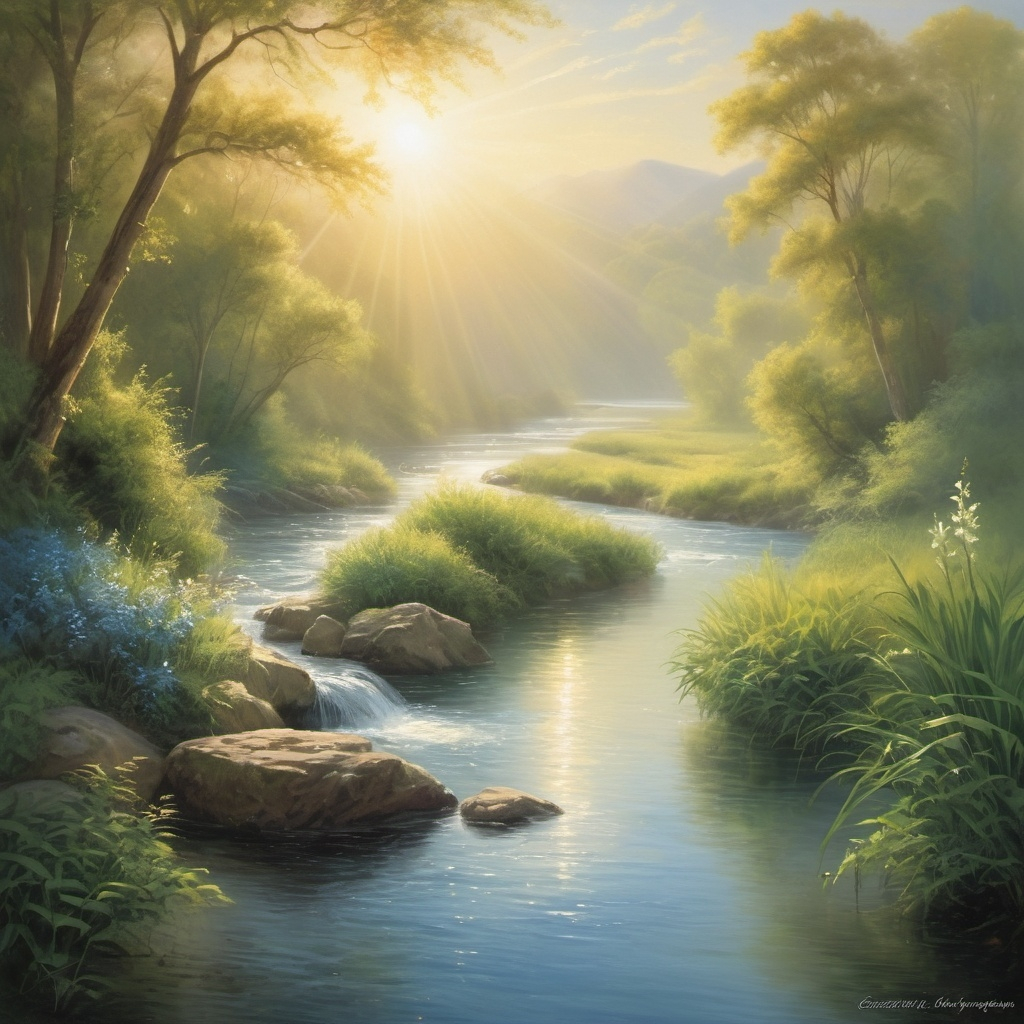


**Recommended Uses**

1. This workbook is intended for self-identified Christians (or someone familiar with Christianity) who may be struggling to experience forgiveness from God.

2. This workbook takes the Bible as its primary authoritative text on the topic of forgiveness from God. It is intended to be complementary to (and not a replacement of) the Bible.

3. If you are part of a denomination with clear guidelines for dealing with sins—such as confession, contrition, and penance—this workbook assumes you have already followed the relevant guidelines.

4. Once you’ve completed this workbook, it can serve as a reference point to reflect on and evaluate the changes that have occurred in your life since you completed it.

5. Because there is no limit to the number of times this workbook can be completed, it can serve as a life-long resource.

6. This workbook is not intended to replace spiritual care from clergy or treatment from qualified professionals such as mental health counselors. If you find that you are experiencing intense struggles around spiritual matters, you are encouraged to seek additional support.

***Note*:** This workbook is intended to be used as a Microsoft Word document in which you type your responses, but you may also complete a hard-copy version by printing the workbook. Please go through the workbook using the instructions that are provided.

**Setting the Stage**

**What is the RECEIVE Forgiveness workbook all about?**

Forgiveness is something all of us need. Whether we are the one offering forgiveness or the one receiving it, we have all formed opinions or views about what forgiveness is and when it is appropriate to offer or accept it. The RECEIVE Forgiveness workbook, however, is not about everything surrounding forgiveness. It is instead about the *experience* of forgiveness. And it’s not just any case of forgiveness. We’re focused on your experience of forgiveness from God for wrongdoing, both wrongs you may have committed by doing something you shouldn’t have done (sins of commission) and by *not* doing something you should have done (sins of omission). The workbook is intended for a broadly ecumenical Christian audience.

From the start, it is very important to note that this workbook won’t get you God’s forgiveness. God’s forgiveness isn’t something we earn. That is, it’s not part of some transaction in response to good behavior. From the theological perspective informing this workbook, forgiveness isn’t earned at all by us mere humans. Rather, God’s forgiveness is always available to those who are contrite. It is just that sometimes we struggle to *experience* God’s forgiveness even though it is already on offer. If you are going through this workbook, we assume that God has already forgiven you. But this workbook will help you to experience that forgiveness, to rest in God’s love of you. In other words, while it is common for Christians to *know* that God forgives them, there are times when people struggle to experience God’s forgiveness. The RECEIVE Forgiveness workbook is intended to help bridge this gap.

The RECEIVE Forgiveness workbook is an opportunity to reflect on your own story of forgiveness from God. Hopefully, you’ll think hard about how God’s love relates to his forgiveness of sin. We hope you’ll experience the freedom and positive transformation that felt divine forgiveness brings. It is also an opportunity to grow spiritually, to meditate on virtue, and to pray for the Holy Spirit to manifest it in your life. The letters of RECEIVE form an acronym that represents the journey through which the workbook will take you:

- **R**emember God’s love for you
- **E**xplore how God forgives
- **C**onfront your sin
- **E**ngage with God to make amends
- **I**nternalize God’s forgiveness
- **V**enture toward renewal and virtue
- **E**stablish a plan to keep experiencing God’s forgiveness

We’ll explore each part further as you progress through the workbook. We have designed the workbook so that the parts of RECEIVE work together to take you on a transformative journey toward a fuller experience of God’s forgiveness.

**What is expected of you?**

This workbook is your GPS for a personal journey. You will complete practical exercises that will help you to engage your own sin and work toward a fuller experience of forgiveness from God. The choice to take the journey is entirely yours. Here are some considerations if you decide to trek through this workbook:

- Each part of this workbook builds on those that came before. So, it is important to complete each part in order. Please don’t skip any exercises.
- By design, a single exercise doesn’t take much time to complete. So, it shouldn’t be too difficult to work the exercises into your schedule.
- You can complete the workbook at your own pace, but you will get the most out of this workbook if you regularly set aside time to complete it.
- You are likely to benefit the most if you complete the entire workbook.

**What can you expect from completing the workbook?**

It is important to note that the RECEIVE Forgiveness workbook offers just one pathway or resource to help in your struggle to experience forgiveness from God for sin. Completing the workbook may bring more benefits to some people than others, and although we have developed this workbook by drawing on rich insights from history, psychology, philosophy, and theology, we cannot promise that this workbook will fully address your struggles. Based on what we have learned, we think this workbook could help you experience meaningful change. For some people, this change might be small. For others, it may be more dramatic. In other words, this workbook may just be the start of your journey to more fully experiencing God’s forgiveness. It also might be the very resource you needed to come “unstuck” on a journey that began much earlier. But wherever you are on your overall journey, we hope that you will approach the RECEIVE Forgiveness workbook with an open heart and mind.

**Before Getting Started**

The RECEIVE Forgiveness workbook includes some text from scripture using the New Revised Standard Version, updated edition (NRSVue) of the Bible unless otherwise noted. If your preferred version of the Bible is different, please feel free to read the verses or passages in that version.

Each time you open up the workbook after setting it aside, we suggest that you try to minimize potential distractions. For instance, you might turn off your cell phone so that you can fully concentrate on what you’re experiencing.

We encourage you to go through this workbook at a pace that is comfortable for you. There are different ways people approach this workbook. Some complete several whole lessons in one session before coming back to the workbook a few days later. Others spend a few minutes on the workbook each day by completing an exercise or two at a time.

Regardless of how you decide to complete the workbook, each time you open it, try to prepare your heart and mind for what you are about to work through. One way we can do this is through prayer.

Before you begin the first lesson, we invite you to read (or pray aloud) one or more of the following prayers. Each time you open the workbook after setting it aside, we encourage you to begin by reading or praying one or more of these prayers before moving into the workbook activities.

1. **The Lord’s Prayer (Matthew 6:9b-13)**

Our Father in heaven,

may your name be revered as holy.

May your kingdom come.

may your will be done

on earth as it is in heaven.

Give to us today our daily bread.

And forgive us our debts,

as we also have forgiven our debtors.

And do not bring us to the time of trial,

but rescue us from the evil one.

1. **Prayer for the Right Use of Gifts (Book of Common Prayer)**

Almighty God, whose loving hand hath given us all that we
possess: Grant us grace that we may honor thee with our
substance, and, remembering the account which we must one
day give, may be faithful stewards of thy bounty, through
Jesus Christ our Lord. Amen.

1. **Psalm 23 – The Lord is My Shepherd (English Standard Version)**

The Lord is my shepherd;

I shall not want.

He makes me lie down in green pastures;

he leads me beside still waters;

He restores my soul;

He leads me in the paths of righteousness

for his name’s sake.

Even though I walk through the valley of the shadow of death,

I will fear no evil;

for you are with me;

your rod and your staff, they comfort me.

You prepare a table before me in the presence of my enemies;

you anoint my head with oil;

my cup overflows.

Surely goodness and mercy shall follow me

all the days of my life;

And I shall dwell in the house of the Lord forever.

**Remember God’s Love for You**

**Lesson 1 – What does it mean for God to love you?**

Scripture teaches us both that God is love and that God loves us. While the idea that God is love might sound obvious, it is worth stopping to ask yourself: “What does it mean to say that God loves *me*?” Scripture tells us that God desires your good—God wants you to have a good life in the fullest sense—and that God wants the deepest friendship possible between himself and you. In fact, God *made* us to be in friendship with him. In that state we will be our happiest, fullest selves. Another way of saying this is to say that our deepest desire is to love God.

Given that God wants deep, true friendship with you which satisfies your deepest longings, you can expect God to act in ways that sync up with his desires for us. In other words, because God loves, we can expect God to act in accordance with that love. Scripture gives voice to this in Romans 5:6-8 where we read:

For while we were still weak, at the right time Christ died for the ungodly. Indeed, rarely will anyone die for a righteous person—though perhaps for a good person someone might actually dare to die. But God proves his love for us in that while we still were sinners Christ died for us.

Sometimes people doubt God’s love for them. Perhaps they think they are insignificant in the grand scheme of things. Psalm 8 gives voice to this when the psalmist rhetorically asks, “What are humans that you are mindful of them?” Other people worry that their sins are so great that God can’t possibly love them.

But as the passage in Romans reminds us, all people—every one of us—are deeply loved by God. He knows who they have been, who they are now, and who they can yet be. God does not love our sins, but he loves the sinner. God loves each of us and because of that love refuses to let sin prevent a loving relationship.

**Exercise 1.1**

Take a few minutes to reflect on God’s love by slowly reading the following scripture passage. Then write out what parts stand out to you most, and why:

For I am convinced that neither death, nor life, nor angels, nor rulers, nor things present, nor things to come, nor powers, nor height, nor depth, nor anything else in all creation will be able to separate us from the love of God in Christ Jesus our Lord (Romans 8:38-39).

| **The parts of this scripture passage that stood out to me:** |
| --- |
|  |

**Exercise 1.2**

In this exercise, you will recite a prayer while changing the emphasis each time you say it. After you have read through these instructions, you are invited to close your eyes in prayer.

The prayer is “God is love”. Each time you repeat it aloud you will change the emphasis, like this:

*God* is love

God *is* love

God is *love*

Take your time as you recite each line. Once you have recited all three emphases, we encourage you keep your eyes closed and slowly repeat the three-line prayer a few more times.

****You may close your eyes and begin the prayer****

What thoughts, feelings, or physical sensations did you experience as you recited this prayer?

| **What I experienced when reciting the prayer:** |
| --- |
|  |

As you alternated between the three emphases, which emphasis affected you the most? Why do you think this was the case?

| **The emphasis that affected me the most:** |
| --- |
|  |

**Lesson 2 – How is God’s love manifest in your life?**

In Lesson 1, we discussed how God’s love for us is made up of two desires: (1) a desire for our good and (2) a desire for deep friendship with us. To really appreciate God’s love for us personally, though, it is sometimes helpful to remember, as written in the book of James: “Every generous act of giving, with every perfect gift, is from above, coming down from the Father of lights, with whom there is no variation or shifting shadow” (1:17).

One thing that is easy to miss in this verse is that it is not only gifts—like freshly baked bread from a neighbor or a birthday present—but generous *acts* themselves that are said to be from God. In other words, whenever we receive something as a gift from another person (a friend or even an acquaintance), besides thanking them for the gift, it is right to thank God twice—for the gift and for the *giver* that in some sense God has sent your way.

**Exercise 2.1**

As we go about our lives, we often get lost in the “things we have to do” to get through the day. It can be easy to lose sight of just how many good things we receive from God and others. In this exercise, you are invited to push against that tendency.

After you have read through the following instructions, close your eyes and begin the first part of this exercise. We suggest you set a timer for five minutes to allow you to settle into this reflective exercise.

Spend some time reflecting on your favorite moments in your life, the experiences in your life that you are most grateful for. Try to imagine that you are there again. What were you doing? Who else was with you? What sounds and smells do you remember from these experiences?

****You may close your eyes and begin the exercise****

God was present in those moments. Take a few minutes to think back on those experiences and reflect on how God was there with you. In the box below, write how you think your favorite moments reveal God’s love for you.

| **How my favorite moments reveal God’s love for me:** |
| --- |
|  |

Let’s take a moment to thank God, the “Father of lights”, for illuminating these good memories for you. Use the box below to make a list of reasons why you are grateful to God for the treasured moments, people, or experiences in your life.

| **Reasons I am grateful to God:** |
| --- |
|  |

We now invite you to pray to God. Prayer can be as simple as talking to God the way you might talk to a friend. God yearns to have you talk to him. After closing your eyes and taking a moment to settle your thoughts, express to God your gratitude for the treasured moments, people, or experiences in your life.

****You may begin your prayer****

**Exercise 2.2**

Read the following quote attributed to St. Thérèse of Lisieux:

“Everything is grace; everything is the direct effect of our Father’s love—difficulties, contradictions, humiliations, all the soul’s miseries, her burdens, her needs—everything, because through them, she learns humility, realizes her weakness. Everything is a grace because everything is God’s gift”.

Use the box below to summarize this quote in your own words (there are no right or wrong answers).

| **My understanding of this quote:** |
| --- |
|  |

In this quote, Thérèse of Lisieux paints the picture of someone striving to find the good in the least likely places. She begins with an unqualified claim that *everything* is grace, all gifts from God. But then she anticipates the cynic who says, “Really, Thérèse? Even my daily miseries? Even my humiliations, shortcomings, and burdens?” Rather than respond to the cynic by adding qualifications, she digs in her heels and says, “Yes, even those things can be seen as gifts by those who learn humility through them”. This is because God is always present with us. God is there in our highest and lowest moments to guide us. God never abandons us. As the psalmist says in Psalm 139:

^7^ Where can I go from your spirit?

Or where can I flee from your presence?

^8^ If I ascend to heaven, you are there;

if I make my bed in Sheol, you are there.

^9^ If I take the wings of the morning

and settle at the farthest limits of the sea,

^10^ even there your hand shall lead me,

and your right hand shall hold me fast.

No matter where we are, God is there. In the heights of heaven or the depths of woe, God is there with us ready to take our hand and lead us, encourage us, or comfort us—whatever we really need. And it is because of this knowledge that the psalmist confidently puts a request to God:

^23^ Search me, O God, and know my heart,

test me and know my thoughts.

^24^ See if there is any wicked way in me,

and lead me in the way everlasting.

Both the psalmist and Thérèse speak of recognizing everything as a gift. Every moment—misery and victory alike—is an opportunity to grow in one’s friendship with the loving God.

Read the quote from St. Thérèse of Lisieux once again. After you have taken a few moments to reflect, use the box below to explore how this quote might apply to your own life. You may find it helpful to consider the following questions as you write:

- Are there parts of her claim that you have trouble believing? If so, can you still thank God that he never forsakes us (Deuteronomy 31:6; Hebrews 13:5) and that he can bring positive meaning out of even the most difficult parts of life?
- What aspects of this quote resonate positively with your experiences in life?
- What experiences have helped you learn that God’s grace is here for everyone and in everything?
- What do you think your life might be like if this outlook were to color the way you see everyday life?

| **How this quote applies to my own life:** |
| --- |
|  |

**Explore how God forgives**

**Lesson 3 – Forgiveness is part of God’s loving nature**

For you, O Lord, are good and forgiving,

abounding in steadfast love to all who call on you.

— Psalm 86:5

Psalm 86:5 tells us that God is forgiving; not just that God forgives, but that God *is characterized by forgiveness*. Readiness to forgive is part of God’s very nature. But the psalm also connects this to God’s love, which we’ve learned amounts to the desires that God has for our good and deep friendship with us.

It is common for lots of us who struggle to experience forgiveness from God to think that we are just too sinful for God to forgive us. But if God’s forgiveness really comes out of his love, consider some wisdom from Bernard of Clairvaux. He wrote that the measure of love is love without measure.^[[1]](#footnote-2)^ In other words, there is no limit to God’s love. It follows then that there is no limit to God’s grace. Both love and grace, forgiveness and gift, are poured out from God into us as vessels that receive as much of these things as we are capable of receiving.

As we explore God’s forgiving character, note that we are not trying to increase God’s love. There is no need to do so. Remember what we just read: God’s love is without measure. Therefore, it is always at the max. It is infinite.

Rather than attempt to increase God’s love (which is impossible anyway), as we explore God’s forgiveness, focus instead internally. With God’s help, we are trying to increase our own capacity to receive God’s love.

**Exercise 3.1**

This activity can help demonstrate the infinite nature of God’s love for us. After you have read the following instructions, you will be prompted to begin the activity. You may want to keep these instructions close by so that you can refer to them if you need to.

1. **Find and take two different-sized drinking cups to a sink.**
2. **Turn on the water faucet.**
   - - This represents God’s love for you and all of creation. It is endless and continues to flow.
3. **Fill the smaller cup to the brim.**
   - - This represents us receiving God’s love early in our walk of faith.
4. **Pour the water from the smaller cup into the larger one.**
   - - This illustrates that as we mature in our faith, our capacity for receiving God’s love increases.
5. **Now fill the larger cup to the brim with the water from the faucet.**
   - - Notice that both cups are easily filled to the brim with water from the faucet. This is what God’s love is like. It always fills us to our maximum capacity for receiving. As our capacity grows, so likewise does the love we receive from God grow.

****You may now begin the activity****

In what ways did this activity get you thinking or feeling differently about God’s love?

| **Ways that this activity got me thinking or feeling differently about God’s love:** |
| --- |
|  |

**Exercise 3.2**

One of the surprising things about scripture is how often it tells us about people who have done things that might seem unforgivable. What’s even more surprising still is how the scriptures describe these same characters.

Consider David, the shepherd boy documented in the Old Testament. After defeating Goliath, the Philistine giant, David eventually became the new king of Israel. David is noteworthy for being described as “a man after [God’s] own heart” (1 Samuel 13:14). Indeed, we see David’s heart on display in many of the psalms that he is reputed to have written. Consider, for example, these lines from Psalm 51:

^10^ Create in me a clean heart, O God,
 and put a new and right spirit within me.
^11^ Do not cast me away from your presence,
 and do not take your holy spirit from me.
^12^ Restore to me the joy of your salvation,
 and sustain in me a willing spirit.

Let’s pause for a moment. Why would someone described as having *a heart after God’s own heart* ask to have that same heart replaced, but this time with a clean heart? Is a heart after God’s own heart not already a clean heart? What could that mean?

What’s important to understand here is that the reason for David’s writing of Psalm 51 is traditionally identified with the narrative describing his exploitation of Bathsheba and subsequent murder of her husband, Uriah (one of David’s loyal soldiers). These actions of David are among the most severe of sins, and they were a failure on his part to love and respect all people involved.

Psalm 51 emerges from this story as David’s heartfelt apology to God. While David is described as a man after God’s own heart, this psalm highlights the tension between his desire to follow God and his capacity to stray. Reconciling this tension requires recognizing David’s *deepest* desire to align his will with the ways of God so that, even when he sins severely, this deepest desire persists and grounds the truth that he is a man after God’s heart.

Despite this, David’s conflicting desires—such as his intention to make advances toward Bathsheba—revealed the “unclean” parts of his heart. David’s prayer for a clean heart, then, is a plea for God to unify his heart’s desires with his deepest commitment to God’s will. In other words, if someone genuinely desires to seek God’s will, like David, they reflect a heart after God. While they may still fall into sin, as David did, they can seek forgiveness because God’s love and grace extend to all sins.

If we apply the water faucet metaphor in Exercise 3.1 to the story about David, one way we can think about David’s sin is that it represents a moment where the overflowing cup that has been filled by the endless supply of water has shrunk; its capacity to receive that water has been momentarily diminished. The solution to this, though, is not to ignore the sin. Ignoring it would leave us divided against ourselves, in a sense hiding from what we have done.

Instead, we should be unified within ourselves, recognizing and confronting our sin. Once we do this, we can be honest with God and present ourselves to God as we are. And in presenting our genuine selves to God, our whole being becomes more open to receiving the loving guidance of God. We open up even our shortcomings, which we have now owned, to his healing power. And in so doing, our capacity to receive the love of God is renewed and even increased so that in our gratitude we are filled even more with God’s freely flowing love.

**Exercise 3.3**

In this exercise, you will read the Parable of the Prodigal Son from Luke 15:11-32, and then answer some reflection questions. Regardless of how familiar you are with this story, we encourage you to read through the passage slowly. Try to be attentive to how the words touch your heart and make you feel. Pause on any words or phrases that impact you deeply, noting how they make you feel and what they stir inside of you.

The Parable of the Prodigal Son (Luke 15:11-32)

^11^Then Jesus said, “There was a man who had two sons. ^12^The younger of them said to his father, ‘Father, give me the share of the wealth that will belong to me.’ So he divided his assets between them. ^13^A few days later the younger son gathered all he had and traveled to a distant region, and there he squandered his wealth in dissolute living. ^14^When he had spent everything, a severe famine took place throughout that region, and he began to be in need. ^15^So he went and hired himself out to one of the citizens of that region, who sent him to his fields to feed the pigs. ^16^He would gladly have filled his stomach with the pods that the pigs were eating; and no one gave him anything. ^17^But when he came to his senses he said, ‘How many of my father’s hired hands have bread enough and to spare, but here I am dying of hunger! ^18^I will get up and go to my father, and I will say to him, “Father, I have sinned against heaven and before you; ^19^I am no longer worthy to be called your son; treat me like one of your hired hands.”’ ^20^So he set off and went to his father. But while he was still far off, his father saw him and was filled with compassion; he ran and put his arms around him and kissed him. ^21^Then the son said to him, ‘Father, I have sinned against heaven and before you; I am no longer worthy to be called your son.’ ^22^But the father said to his slaves, ‘Quickly, bring out a robe—the best one—and put it on him; put a ring on his finger and sandals on his feet. ^23^And get the fatted calf and kill it, and let us eat and celebrate, ^24^for this son of mine was dead and is alive again; he was lost and is found!’ And they began to celebrate.

^25^ “Now his elder son was in the field, and as he came and approached the house, he heard music and dancing. ^26^He called one of the slaves and asked what was going on. ^27^He replied, ‘Your brother has come, and your father has killed the fatted calf because he has got him back safe and sound.’ ^28^Then he became angry and refused to go in. His father came out and began to plead with him. ^29^But he answered his father, ‘Listen! For all these years I have been working like a slave for you, and I have never disobeyed your command, yet you have never given me even a young goat so that I might celebrate with my friends. ^30^But when this son of yours came back, who has devoured your assets with prostitutes, you killed the fatted calf for him!’ ^31^Then the father said to him, ‘Son, you are always with me, and all that is mine is yours. ^32^But we had to celebrate and rejoice, because this brother of yours was dead and has come to life; he was lost and has been found.’”

Reflecting on your life at present, which person in the story do you find yourself relating to the ***most***, and why?

| **Person in the story I relate most to:** |
| --- |
|  |

What words or phrases caused you to pause, if any? How did they make you feel? Why do you think these words or phrases elicited such a reaction in you?

| **Words or phrases that elicited a reaction in me:** |
| --- |
|  |

**Exercise 3.4**

In the late 1600s, Rembrandt painted the “Return of the Prodigal Son”, which depicts the moment the prodigal son returns to his father. A copy of this painting can be found below. We invite you to find a comfortable sitting or standing position where this picture of the painting is directly in front of you or positioned in a way that makes it easy for you to view it for several minutes. When you are ready, spend some time viewing the painting in light of the passage you read in Exercise 3.3. You can explore and reflect on any aspect of the painting that interests you. You may find it helpful to set a timer for five minutes so you can settle into this reflective exercise.


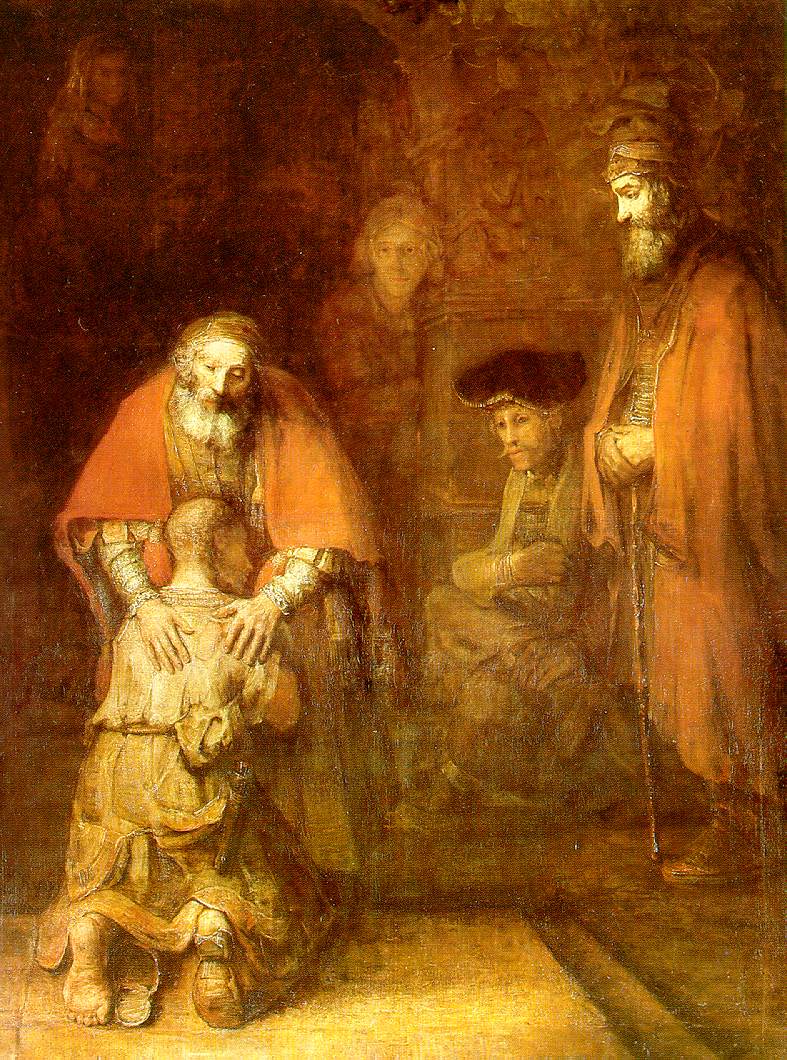


*Return of the Prodigal Son*, Rembrandt

What thoughts, feelings, or physical sensations did you experience in the few minutes you spent viewing and reflecting on this painting?

| **The thoughts, feeling, or physical sensations I experienced:** |
| --- |
|  |

Try to imagine for a moment that you are the prodigal son in the parable. How would you describe what the prodigal son is likely experiencing in this painting?

| **What the prodigal son is likely experiencing:** |
| --- |
|  |

As the prodigal son prepared to return to his father, we can imagine him reflecting on questions like “What will my father do when I arrive?” or “What will he say to me?” His expectations were low. He hoped, at best, that his father would respond by “treating [him] like one of [his] hired hands” because he felt unworthy to be his son. We can infer from the passage that the prodigal son would have been satisfied with this outcome. After all, he knew that being treated as a hired hand in his father’s house would have been better than his own life before returning to his father. Can you imagine how he felt when he saw his father running to meet him, embracing him, and calling for a celebration to mark his son’s return?

The prodigal son expected that, upon returning to his father, he would be treated as a hired hand. But imagine the prodigal son’s surprise when his father met him with a hug, a fresh wardrobe, and a feast in celebration. What do you think the prodigal son learned at that moment about his father’s love for him?

| **What the prodigal son likely learned about his father’s love for him:** |
| --- |
|  |

The Parable of the Prodigal Son provides a glimpse into God’s love for us. God’s love far exceeds our own expectations. The parable also reminds us that no matter what we have done, no matter who we are, no matter how far we think we have fallen, the *fullness* of God’s loving forgiveness for us remains on offer.

**Lesson 4 – God’s forgiveness is available to each of us**

God’s forgiveness is an extension of God’s love. It is always on offer to us. But sometimes we can struggle to experience God’s forgiveness because our sin seems too problematic or unworthy of God’s forgiveness. In this lesson, we look more closely at how God’s forgiveness is available to all of us, all of the time, for any and all our sins.

**Exercise 4.1**

In Luke 19:1-10, the author recounts the story of a man named Zacchaeus who lived in Jericho and was known for his wealth as a tax collector. He was not just any tax collector. He was also Jewish, a *chief* tax collector, presumably *stealing* more than his fair share of taxes from those in his region, and working for the Roman government.

This last aspect of Zacchaeus’ life—the fact that he worked for Rome—was particularly troubling to his friends and neighbors, for Rome was viewed as an enemy of the Jewish people. And to work for Rome, to be a tax collector, was seen by many Jews as being a sell-out, a traitor to one’s people. When you add this cultural fact to the theft Zacchaeus was carrying out at others’ expense, it is easy to see why Zacchaeus was unpopular.

When Jesus entered Jericho, Zacchaeus wanted to see Jesus but was unable to do so since he was a short person in a large crowd. Zacchaeus, however, showed both his knack for problem-solving and his desperation to see Jesus by finding a tree to climb so he could catch sight of Jesus as he passed through. This was clearly an act of desperation because, in that culture, almost no one of Zacchaeus’ economically privileged status would ever have been caught climbing a tree like a child.

Think about Zacchaeus and the measures he took to see Jesus. Why do you think he was so interested in seeing Jesus?

| **Reason/s I think Zacchaeus was interested in seeing Jesus:** |
| --- |
|  |

It is interesting that the author of Luke doesn’t tell us Zacchaeus’ motivations for seeing Jesus. He may have wanted forgiveness, yes, but he may have just been curious as well. Jesus was something of a celebrity at this point, and Zacchaeus may have simply wanted to add Jesus’ name to the list of influential people he’d met or seen.

Although we don’t know Zacchaeus’ motivations for wanting to see Jesus, Jesus didn’t wait for those motivations to reveal themselves. Before Zacchaeus even introduced himself from atop the tree, Jesus looked at him and called him by name, saying, “Come down, Zacchaeus, because I need a place to stay…salvation has come to your house” (Luke 19:5 & 9). Even someone like Zacchaeus, whom we can assume would have been deemed unworthy of forgiveness according to the Jewish people, had forgiveness available to him through Jesus even *before he asked*.

Take a moment to reflect on Jesus’ motives for forgiving Zacchaeus. Why do you think he forgave Zacchaeus even before Zacchaeus asked to be forgiven?

| **Reason/s I think Jesus forgave Zacchaeus:** |
| --- |
|  |

**Exercise 4.2**

In Jewish culture around the time of Jesus, people like Zacchaeus were considered some of the greatest sinners. Although our modern culture is somewhat different, our laws suggest that some sins are more severe than others. We can tell because the penalty incurred by them is greater. In this activity, you are going to imagine that you have received a handwritten letter from someone in prison who has committed a crime that carries a prison sentence. In this letter, the person tells you what they did, how it was a sin, how sincerely sorry they are, that they have come to a life of faith in Jesus, and how they’ve committed to live differently. In the last part of the letter, they open up about their struggles with experiencing God’s forgiveness because they don’t see how God could ever forgive them for what they did.

Use the box below to write a brief letter to this person to explain how God’s forgiveness is available even to them. Before you write the letter, take a moment to think about some of the events from scripture we have discussed: the interaction between Jesus and Zacchaeus, David’s confession to God, or the parable of the prodigal son. Consider including lessons from those stories in your letter.

| **My letter to the person who is struggling to see how God could forgive them:** |
| --- |
|  |

**Confront your sin**

**Lesson 5 – Where do I begin the journey of addressing the sin I am struggling with?**

Sin has been defined in various ways. In Greek, the New Testament word translated as sin is *hamartia*, which means “missing the mark”—like when an archer fails to hit a bullseye. This imagery captures how we fall short of God’s desires for our lives when we sin. Sometimes we miss high. Sometimes we miss low. And sometimes we are just barely off center. But all are different ways of missing the goal that we can work to correct.

Other definitions of sin focus on the image of God as lawmaker, with sin defined as transgressing God’s law. Such definitions usually remind us that the laws God gives are given out of love for us. Acting in line with God’s law brings about our greatest flourishing. For if God loves us, then God’s decrees over us will be aimed at our ultimate good. Because this workbook takes God’s love as primary, it draws upon a definition of sin that emerges from God’s enduring love for us.

Scripture reveals that God’s love is steadfast and eternal. We saw this in the story of David, the lessons from the parable of the Prodigal Son, and the way that Jesus interacted with Zacchaeus. But because of our human condition, we sometimes fail to love God and others fully. These shortcomings are sins, that is, moments of turning away from God. This can manifest as either “sins of commission”—times when we *act* wrongly—or “sins of omission”—when we *do not act* though we know we should.

Confronting sin requires more than simply identifying what we did (or didn’t do) and calling it a day. This is because our actions, whether good or bad, often flow out of our deeper insecurities or misplaced desires as human beings. For example, dishonesty on a job resumé might reveal a sense of entitlement—pride in thinking we deserve a job regardless of merit. Or maybe we don’t give up our bus seat to someone else who needs it because we are so consumed in social media on our phone that we lack the presence of mind to consider budging from our seat.

Theologians have long explored the root causes of sin. Saint Augustine, whose writings have influenced huge swaths of theology (perhaps more than any other theologian), claims that all sin emerges from *pride*, from putting our own interests or comforts before those of others. Other theologians, like the late Tim Keller or the Reformer John Calvin, suggest that *idolatry*—placing something created above God—underlies all sin. Both of these perspectives have strong historical grounding and biblical support.

But rather than choosing between these views, it may be more helpful to reflect on how *both* pride and idolatry shape our own struggles. There’s no doubt, after all, that they are at least involved much of the time, and if we reflect on our own sins from either perspective, this can help us better understand how the ways we think of ourselves and God might lead us into sin.

**Exercise 5.1**

You spent the first four lessons of the workbook reflecting deeply on God’s steadfast love and forgiveness. While keeping that love and forgiveness at the front of your mind, we now invite you *to recall a specific sin* for which you're struggling to feel God’s forgiveness (that is, a time you turned away from God or transgressed God’s law). We recognize that this can be an uncomfortable and deeply personal experience. Try to hold in your mind and heart God’s love while you think of the sin. You will be asked to think about this specific sin at different points throughout the remainder of the workbook. These reflections are not intended to be judgments or to trigger self-condemnation. Rather, they are opportunities to enhance your experience of God’s forgiveness.

Please take a moment to bring this specific sin to mind. When you are ready, write this specific sin down in the box provided below. Acknowledging our sin with honesty can be challenging, but it can be an important step in learning, growth, and deepening our relationship with God.

| **The sin for which I’m struggling to experience forgiveness from God is:** |
| --- |
|  |

Take a deep breath and give yourself credit for acknowledging and writing down this sin.

**Exercise 5.2**

Theologian Reinhold Niebuhr challenges us to imagine we are sailors climbing a ship’s mast. At the bottom of the mast lies the safety of the ship’s main deck, a place of comfort and familiarity. At the top is the crow’s nest, where one can see the vast expanse of the ocean—an exhilarating experience of transcendence. From this height, the view is breathtaking.

Niebuhr suggests that in our struggle with sin, we are like sailors caught mid-climb, suspended between “the ‘abyss’ of the waves beneath and the ‘crow’s nest’ above.”^[[2]](#footnote-3)^ As we ascend, the dizzying sight of the sea far below stirs our fear and anxiety, tempting us to descend—to retreat back to the security of the deck and the comforts of earthly life. And yet, as we climb higher, we begin to sense our freedom—the astonishing capacity we have been given to transcend, to know, to act. We are impressed with ourselves, with the strength and vision granted to us.

In Niebuhr’s terms, we are caught between our finitude and our freedom, our fear and our pride, and from this tension, our sins emerge.

Imagine you are the sailor climbing the mast that Niebuhr describes. The mast is represented in the picture below by the solid vertical line. When you think of the sin you wrote down in Exercise 5.1, are you being drawn toward the top part of the mast, drawn to pride and excessive self-reliance? Or are you being pulled toward the bottom part, where worldly distractions are to be found? Or are you being pulled in both directions, yo-yo-ing in-between?

On the left side of the picture, there is a red “**X**”. Click on and move the red “**X**” to mark where you feel you are on the line as it relates to the specific sin you wrote about in Exercise 5.1. If you are completing a printed version of this workbook, use a pen or pencil to mark your “X” on the line.

**X**

Using the box below, describe why you put the “**X**” on that specific location on the mast? Does this particular sin feel like it is a response to freedom, finitude, or a combination of both?

| **Reason/s I put the “X” on that specific location:** |
| --- |
|  |

**Exercise 5.3**

Psalm 32:5 describes the practice of confessing the full extent of one’s sin directly to God: “Then I acknowledged my sin to you, and did not hide my iniquity”. Some people might be tempted to downplay the need to confess sin to God because God already knows what they have done and is ready to forgive. But remember our discussion in Lesson 3 of David’s confession in Psalm 51. Confessions are, in large part, about healing *us*. They are a way of increasing our capacity to receive God’s love and realign our will with God’s will.

With this in view, we invite you now to confess with the following traditional prayer of contrition. **Read it and see whether you can agree with it. Then recite it out loud in sincerity:**

My God, I am sorry for my sins with all my heart. In choosing to do wrong and failing to do good, I have sinned against you whom I should love above all things. I firmly intend, with your help, to do penance, to sin no more, and to avoid whatever leads me to sin. Our savior, Jesus Christ, suffered and died for us. In his name, my God, have mercy.

**Exercise 5.4**

The prayer of contrition above is very general so that virtually any Christian can say it as applied to sins they may have committed. This is helpful as an exercise in its own right because, in saying that prayer, we know that there are millions of other Christians around the world saying it with us in solidarity. But even so, it can also be helpful to be more specific, too.

In the box below, we have reproduced the beginning of the prayer of contrition followed by a blank **orange** space where you can fill in the specifics of the sin you wrote about in Exercise 5.1. This is your time to talk to God about the sin. So, in that orange space, while being reasonably detailed and honest, state (1) what happened and (2) how your actions transgressed God’s standards or values. Remember, you are writing this letter to the “Father of Lights” (James 1:17) in whom there is no darkness and whose love for you has no bounds.

For guidance, here is an example using the story of Zacchaeus we explored earlier:

My God, I am sorry with my whole heart for *taking advantage of my professional position to take money for myself that was not mine to take.*

| **My personal prayer of contrition to God** |
| --- |
| **My God, I am sorry with my whole heart for…** |
|  |

We now invite you to take this personal prayer that you’ve written and read it aloud to our loving God. Before doing so, spend some time considering whether there might be a specific location where you’d like to read it aloud. Is there a particular place that you feel especially connected to God’s loving presence, such as a specific room where you live, a park or trail, or church? If so, we encourage you to speak the prayer aloud to God in that place (if feasible).

What was it like to say your personal prayer of contrition out loud to God? How did it make you feel? Describe your experience using the box provided below.

| **What I experienced as I said my prayer aloud to God:** |
| --- |
|  |

**Lesson 6 – Explore Sin’s Impact to Identify Places for Growth**

Sin involves turning away from God, yet God is always loving and loves us despite our sinfulness. Moreover, God differentiates between the sin and the sinner: God does not love our sin, but God always loves *us*. In fact, it is *because* of his love for us that God does not love sin.

Now, the deepest desire of a Christian is a desire for God and the goodness that he is. Indeed, in the Christian tradition, this deepest, fundamental desire is said to be found in all humans, even in those who are unaware of it. To put it in the words of Saint Augustine, “our hearts are restless until they rest in You”.^[[3]](#footnote-4)^ The idea is simple: we can pursue all kinds of goods in this world—friendship, food, worldly success, fame—but unless those goods are understood in their proper place with respect to our pursuit of God—in light of our dependence on him—we will be left dissatisfied.

When we sin, we act on a desire for something other than God, something that conflicts with that deepest desire that only God can satisfy. Our sin, then, takes us to a place where we find our own desires undermining each other. In such a state, we are divided against ourselves; stuck in a situation where no matter what we choose, we can’t have everything we desire. Also, because our sinful desires are desires that go against God, we cannot wholeheartedly desire him. Sin, in other words, diminishes our capacity for loving and being loved, and this is part of why God wants to remove it.

So, while sin does have to be dealt with, confronting our sin does not mean we are pushing away God’s love. On the contrary, confronting sin is a process by which we increase our capacity to receive God’s love.

Because God’s love involves his desire for our good, confronting our sin in a healthy way can put us on the path of transformation and growth that aligns with the goodness that God desires for us. This does not mean that the process of confronting our sin is easy. In fact, the discomfort we experience when we confront our sin is real and difficult. This is why we often try to minimize, excuse, rationalize, or justify our sin. Sometimes we avoid confronting our sin altogether. But when we don’t meet our sin head-on, we close ourselves off from experiencing the fullness of God’s love in our lives. While confronting our sin can make us feel vulnerable, if we can take this step it opens us up to the possibility of deeper friendship with a loving God.

In some ways, confronting one’s sin is the most difficult part of our journey toward experiencing more of God’s forgiveness. You have done well to take an important step forward by acknowledging the specific sin in Lesson 5. In this lesson, we invite you to continue pressing forward in confronting this particular sin. Although this may feel uncomfortable or difficult, remember that confronting our sin opens us up to the fullness of God’s love, and that it leads down a path, on which you’re already traveling, to a peace that surpasses understanding.

**Exercise 6.1**

Another important part of confronting our sin is to identify and be honest about the full extent of its impact. From one angle, this might seem fearful and daunting, but from another perspective, we can feel empowered to be accountable for the impact of the sin because (a) we know God’s love for us never changes and (b) we can expect God’s help in correcting many of the difficulties our sin may have created.

In this exercise, we invite you to reflect on the impact that this sin has had on (1) you, (2) other people, and (3) your relationship with God. The purpose of this exercise is not to condemn or shame ourselves for our sin, but to compassionately explore how the sin has affected different aspects of our lives so that we are armed with deeper insights that could benefit our journeys of transformation and growth.

Using the **orange** spaces provided below, briefly describe how the specific sin you wrote about in Lesson 5 is impacting the three areas mentioned above. There are three orange rows for each area, but you can add more rows if needed. We have provided some examples to help guide you, but what you write should reflect what you have experienced.

| **How has this specific sin impacted *you*?** |
| --- |
| *Example 1*: I feel a sense of guilt over what I did. |
| *Example 2*: I have lost confidence in myself because of my actions. |
| 1. |
| 2. |
| 3. |

| **How has this specific sin impacted *others*?** |
| --- |
| *Example 1*: I damaged my relationship with my friend because of the hurtful things I said to them. |
| *Example 2*: I have deeply hurt my partner. |
| 1. |
| 2. |
| 3. |

| **How has this specific sin impacted *your relationship with God*?** |
| --- |
| *Example 1*: My bond with God has been disrupted by this sin. |
| *Example 2*: It feels like God is punishing me for what I did. |
| 1. |
| 2. |
| 3. |

**Exercise 6.2**

Another important step in confronting our sin is deciding what to do with it. Consider the following quote:

*Freedom is found when you utilize the lessons learned by your failures as stepping stones toward abundant life.*

- - - - Katherine Walden

As this quote suggests, we need to make use of our failures to love and choose God by *learning from them*. If we are going to learn from our sin, we need to reflect on the underlying motives behind it. During such reflection, we should ask ourselves what led to the sin. Consider the example from Lesson 5 where a person lied on their resumé because they felt entitled to get a job even when they weren’t qualified. In that case, we might say the person lacked honesty, gratitude, or humility: honesty because they didn’t tell the truth when they should have, gratitude because perhaps they were not sufficiently thankful for their current employment and salary (even if it was perhaps less than they merited), or humility because their decision seemed motivated by a sense of self-importance.

Following this example, take a few moments to think about the underlying reasons behind the specific sin you wrote about in your letter to God (Exercise 5.4). What do you think motivated you to sin in this particular way? Use the box below to describe your motives. Try to be as honest with yourself as possible.

| **My motives underlying this specific sin:** |
| --- |
|  |

**Exercise 6.3**

Another part of what it means to learn from our sin involves spending some time reflecting on what we might have done differently to avoid the sin in the first place. What we learn from this reflective process could help us to not turn away from God in the same way in the future. Of course, knowing what we might have done differently does not guarantee we will avoid the same sin in the future. However, it can put us in a better position to maintain alignment with God’s will and to remain in the fullness of God’s love for us.

Sometimes when we sin there are things about our *environment*—external factors—that we could have altered to avoid sinning. For instance, did you allow yourself to be in a situation that you knew would lead to temptation (e.g., returning to the blackjack table despite knowing it would bring out your out-of-control side)? Or did you feel pressure to sin because of the people you were spending time with or the activities you were doing (e.g., scrolling through your social media and feeding your envy or frustration of others in a way that would impact those nearby)?

At other times there are *internal* factors that can make a difference. The apostle Paul writes in Philippians 4:8:

whatever is true, whatever is honorable, whatever is just, whatever is pure, whatever is pleasing, whatever is commendable, if there is any excellence and if there is anything worthy of praise, think about these things.

What Paul is suggesting here is that if we want to set ourselves up to avoid sin and fully experience God’s love, we should do more than avoid tempting situations. We should fill our minds with good, commendable, and excellent things. This could include listening to sermons, spending time with good friends in celebration, or meditating on scripture. You can decide in conversation with God what “whatever is worthy of praise” might be for you.

In this exercise, we invite you to explore what you think you could have done differently to avoid the specific sin you wrote about in your letter to God (Exercise 5.4). After you have taken a few moments to reflect, use the box below to describe what comes to mind.

| **What I could have done differently to avoid this sin:** |
| --- |
|  |

**Exercise 6.4**

Throughout this lesson, you have worked through some challenging material and activities that can bring up a wide range of internal experiences. This is an entirely normal experience as we take time to intentionally confront our sin.

For this exercise, let’s return to Lesson 3 where we used the water faucet metaphor to illustrate how David’s sin momentarily diminished the capacity of his cup to receive the endless supply of water that represents God’s love. While our sin diminishes the capacity of our cup to receive God’s love, the steps you have taken to work through the exercises in this lesson have had the opposite effect—they have *increased* the capacity of your cup to receive God’s love.

Take a few moments to reflect on this: Your progress through this workbook so far has increased your capacity to receive God’s infinite love. How does this make you feel?

| **Emotions I experience when I think about how my progress has increased my capacity to receive God’s love:** |
| --- |
|  |

As you continue with the next lesson, remember that by doing this workbook you are opening yourself up more and more to the goodness that God desires for your life and for the deep friendship God desires with you.

**Engage with God to make amends**

**Lesson 7 – How can I work with God to address my wrongdoing?**

^17^ So if anyone is in Christ, there is a new creation: everything old has passed away; look, new things have come into being! ^18^ All this is from God, who reconciled us to himself through Christ and has given us the ministry of reconciliation; ^19^ that is, in Christ God was reconciling the world to himself, not counting their trespasses against them, and entrusting the message of reconciliation to us (2 Corinthians 5:17-19).

Throughout the previous lessons, we have explored how God always (a) desires good for us and (b) desires deep friendship with us. These are the two desires that make up God’s love for us. We have also recalled a specific sin we are struggling with, thought about what it means for God to love us fully in spite of our sin, and explored the impact our sin may have had on ourselves, others, and our relationship with God.

Fixing what’s gone wrong due to our sin—that is, addressing its impact on oneself, our relationship to God, and others—is the process of *making amends*. This can involve apology, but it also includes an intentional effort on our part to repair any damage brought about by our actions (or inactions) insofar as we are able. Thus, we might make amends by reconciling a broken relationship, paying for damaged goods, or maybe even something a bit more creative such as planting a tree in honor of a friend we’ve wronged who is heavily invested in environmental care. Sometimes, we make amends directly to persons we have hurt, but other times it might be appropriate not to involve the hurt party but rather do good deeds for others or other causes.

Take a moment to consider how you’ve tried to make amends for past offenses.

**Exercise 7.1**

| **How have you tried to make amends in past offenses? How about for *this sin*? (from Exercise 5.4)** |
| --- |
| 1. |
| 2. |
| 3. |

As we explored in Lesson 6, it is not always easy making amends on our own to undo the impact on the world resulting from our sin. Because of this, it is even more crucial that we do not forget God’s role in restoring the world.

Consider a parent with a child who steals and eats a candy bar in full knowledge and awareness that what they are doing is wrong. Suppose that the child has no money whatsoever but that the store manager asks the child how they are planning to pay for it. The problem, of course, is that the child cannot pay herself, for she has no money with which to pay. To right this wrong, to fix what has been thrown off, the child must *rely* on someone else. Suppose the child turns to her parent who offers the store manager the necessary amount of money in her place. This act of the parent in which they fix the wrong for the child is an act of *vicarious amends*.

We, like the child in the story, cannot always make amends for the wrongs we’ve done. Some wrongs, for instance, provoke others to break off their relationship with us, leaving us with no way to even attempt to fix what has gone wrong. But we, like the child, are not left without an advocate, and a resourceful one at that, to offer amends in our place. God is able to transform *all* of creation (“everything has passed away; look, new things have come into being”), to reconcile—which means to put right, to *fix*—everything. Thus, even when we find ourselves faced with the effects of a sin that seem to us irreversible, we must remember that God himself ultimately offers amends vicariously on our behalf, fixing all that is yet to have been put right, and doing so out of parental love for each of us.

**Exercise 7.2**

As we noted near the beginning of this lesson, there are many possible ways we can try to make amends for our sin. Some ways to make amends are more *personal*. For instance, if I find myself responding too quickly in anger toward my friend, I might make amends by offering an apology directly to them and engaging in scriptural meditation in hopes of becoming more patient over time. Other ways of making amends take a more *communal* form. For instance, perhaps in addition to an apology and scriptural meditation, I join an online anger management group where I and others with similar experiences of anger can talk with one another and encourage each other in growing toward Christ-likeness. Indeed, both forms of amends—personal and communal—can be pursued in tandem.

Some options for making amends may be more suitable than others, and it is important that we try to discern what appropriate amends might look like for our sin. In Lesson 6, you identified some of the ways your specific sin impacted yourself, others, and your relationship with God. In this exercise, you are going to *explore* *how you might offer amends* for some of those impacted by your sin and *commit to enacting* at least one of those amends. You have two options from which to choose.

**Option 1**: Identify someone in your life who you can trust and with whom you feel comfortable talking about this particular sin. This person could be a spiritual leader, a close friend, counselor, or a mature family member. Find a time to talk with this person to explore some of the ways you might try to make amends for this particular sin. You may find it helpful to talk with them more than once. *After you meet with them, use the boxes below to record the path/s of amends you plan to pursue.*

**Option 2**: If you prefer to engage in this discernment process directly with God, you can alternatively pray to the Father of Lights to help you determine how you might go about trying to make amends for this particular sin. Either pray the prayer provided immediately below, or, if you prefer, approach God with your own personal prayer. After praying, take as much time as you need to reflect on both personal and communal options for making amends for your sin (we recommend taking at least five minutes for this). *Write your reflections in the box provided below.*

***Prayer for God’s Guidance in Making Amends***

O God, there is no darkness in you. May your light reveal paths of reconciliation and peace to me that I might follow them and mend my relationships with those that I have wronged. May your perfect love cast out all my fear and anxiety and replace those things with courage and humility as I walk the path of righteousness for your name’s sake.

Now that you’ve reflected on appropriate amends either in conversation with a trusted person (if you chose Option 1) or in prayer (if you chose Option 2), use the boxes below to list the amends that you will try to pursue. Some examples to get you started include (a) apologizing to those you have hurt or (b) finding and joining a support group.

| **Appropriate amends for this sin that I plan to pursue:** |
| --- |
| 1. |
| 2. |
| 3. |

**Exercise 7.3**

In this exercise, we are going to take a step forward in our pursuit of making amends. As we noted earlier, one possible amend we can pursue is apology. In Lesson 5 you prayed an apology to God. In this exercise, we are apologizing to others affected by our sin as well, including ourselves. In this exercise, you will write a letter of apology to those who may have been affected by the specific sin you wrote about in Exercise 5.1. Use the box provided below to write your apology letter. This exercise is focused on *writing* an apology, but not yet giving it. Later, we will take time to reflect on whether it is fitting to apologize to others. For now, use the letter to think about the ways you could apologize. Consider these:

- Who are you sorry to, and why are you sorry?
- If other people have been *directly* impacted by this sin, how do you think they feel, or would feel if they knew, about the way/s that this specific sin has affected them?
- Who has been *indirectly* impacted by this sin—perhaps those who love us and those we love?
- How do you feel when you think about the hurt or pain you may have caused others? Try to personalize your expression of empathy for the specific people who have been affected.

| **My apology letter:** |
| --- |
| Dear … |

**Exercise 7.4**

In this exercise, you are invited to read your apology letter aloud. Find a quiet and safe place where no one will disturb you. After you have read these instructions, you are going to read your letter of apology out loud to an audience of one: Jesus. You may begin the exercise when prompted.

Take a few moments to visualize Jesus in the space with you. Where do you picture him? Is he standing in front of you, or perhaps sitting in a chair or on the floor? Picture him quietly in your midst with all of his attention focused on you. As you think of the love Jesus has for you, what is the expression on his face? Find a comfortable standing or seated position near where you are picturing Jesus. Some people prefer to stand, others prefer to sit in a chair or on the floor. Take a few deep breaths to settle your mind.

****You may now read your letter out loud****

Using the box below, briefly describe what it was like to read your apology letter aloud while picturing Jesus there with you.

| **My experience reading my apology letter aloud to Jesus:** |
| --- |
|  |

When we share our letter of apology aloud, we can feel vulnerable. But this can also bring us freedom. We are taking responsibility for our role in the sin we’re struggling with and trying to recognize how our actions may have affected others. By reading your apology letter aloud, you have taken another important step forward in the process of entering more fully into the richness of God’s love for you.

There is another step you may take with your apology letter as well. Because our sin often impacts other people, we may want to apologize *directly* to those affected as a way of trying to make amends. But this step isn’t necessarily something that everyone should take. Sometimes, it may not be possible for us to provide a formal apology to people who have been impacted by our sin, or it may not be the best time for us to share our apology with them directly. Two things can help you decide whether to share your apology directly. First, you might want to talk to someone you trust and get an outside perspective. Second, you will want to consider carefully the likely impact on the person your sin affected.

**Exercise 7.5**

What do you think the possible effects might be if you shared your apology? Name three distinct possibilities. After doing so, consider which is most likely:

| **Possible effects of sharing my apology:** |
| --- |
| 1. |
| 2. |
| 3. |

With these possible effects of sharing your apology in view, consider whether it would be suitable for you to do so (either in whole or in part) with those who have been impacted by your sin. *Before sharing your apology though, we suggest you talk with someone that you trust (e.g., family member, spiritual leader, close friend) about this because they could help you discern whether sharing the apology directly with those who have been affected by this sin is the right decision at the present time.*

If, according to your discretion, it doesn’t seem appropriate to share your apology or it doesn’t feel like the right time to do so, you can always return to this possibility later. If you do decide to share your apology, you’ll want to consider whether you should share the entire letter or just the parts that are relevant to the people you apologize to. You will also need to decide when, where, and what format might be best to share your apology with those affected. You could share your apology in person, over the phone, or over a video call, and you can make it as formal or informal as you would like.

Regardless of whether you share your apology or not, remember the opening verses from 2 Corinthians 5. Because you are “in Christ” you are “a new creation: everything old has passed away; look, new things have come into being” (vs. 17). For you, this means that you are not defined by your former sin. You are defined *by Christ* who is there making amends alongside you, always.

**Internalize God’s forgiveness**

**Lesson 8 – Letting go in order to fully move forward**

If you have found yourself experiencing ups and downs at different points in this workbook, we want to acknowledge the difficult work you have done to turn to God with your sin and work through it. You are not alone in your experience; many people have similar experiences as they go through this workbook. By persevering through these ups and downs, you are opening yourself up to experiencing the fullness of God’s love in your life.

**Exercise 8.1**

We now invite you to participate in a brief meditation. There are two ways you can participate: an audio version or a reading version. After completing either the audio version or the reading version, please move on to the 8.1 Written Exercise.

Audio Version:

If you are completing an electronic version of this workbook using Microsoft Word, you can hit the “play” button for the audio clip below and go through the meditation by following the narrator’s instructions. When you have participated in the meditation, return to complete the written part of this exercise below (8.1 Written Exercise).

[
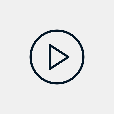
](https://youtu.be/RCZ86Wx0WLw)

[Click Here to Begin](https://youtu.be/RCZ86Wx0WLw) →

Reading Version:

If you experience difficulty opening the audio clip or you are using a printed version of this workbook, you can find the meditation at <https://tinyurl.com/8-1-meditation>. You may also complete the meditation without the audio by following the instructions below.

You will first read some instructions. After you have gone through the instructions, you will see the prompt to begin. When you do begin, try to complete the meditation with your eyes closed. We suggest setting a timer for five minutes so you can settle into it.

*Take a few deep breaths to settle your mind. Then, imagine you are standing in a pitch-dark room that is unfamiliar; you can only see your hands close up. Notice how it feels to be in such darkness.*

*Across the room, faint strips of light seem to outline what appears to be a window partially covered by a dark curtain. You decide that moving toward this potential source of light is your best chance to get your bearings and try to find your way out of the room. You take a step toward it, but your leg bumps into something unseen. As you try to walk around the object, you discover that there are many objects scattered across the floor. Each step you take brings a new and unforeseen challenge, forcing you to navigate carefully. Take note of any emotions that arise as you face these unseen obstacles.*

*Gradually, the light from the window grows clearer. Although it takes time and effort, you eventually make it to the other side of the room within reaching distance of the curtain. You slowly pull the curtain aside. The initial burst of light is overwhelming, but your eyes adjust after a few moments. You look around the room and spot a door near the side of the room you came from. Now that you’re able to see, you take the simplest path to exit the room.*

****You may now close your eyes and begin the exercise****

8.1 Written Exercise:

What thoughts, feelings, or physical sensations did you experience as you completed this meditation exercise? Describe your experience using the box provided below.

| **What I experienced as I completed this meditation exercise:** |
| --- |
|  |

**Exercise 8.2**

The meditation exercise you completed in Exercise 8.1 is a metaphor we can use to explore your journey through this workbook. Let the light from the window behind the curtain represent God’s forgiveness. When we sin, it is as if the light from the window has been blocked because the curtain has been drawn closed by our sin. That doesn’t mean the light has disappeared or no longer exists. God offers forgiveness all the time, even when we have blocked ourselves from fully receiving it.

But like the obstacles faced in the dark room as we imagined ourselves taking steps forward to open the curtain, we may encounter difficulties as we reflect on and process our sin. It can take time and effort to fully overcome the challenges we experience on our way to the curtain, on our way to embracing the light of God’s forgiveness, but by confronting our sin we open ourselves up to fully receive it. Just as drawing the curtain open illuminated the room to reveal the path to the exit door, each step you have taken to confront your sin in this workbook has brought you closer to leaving your sin behind and more fully experiencing God’s illuminating love in your life.

As you reflect on your experience through the workbook so far, which part of this metaphor stands out to you the most and why? There are many different ways in which you might connect with this metaphor, but some examples to get you started include: (a) I noticed that I hold myself back from experiencing the fullness of God’s love in my life when I try to avoid dealing with my sin, or (b) I realized that I was avoiding even looking at the outline of light on the wall. All I could think of was the fact that I had sinned, and it overwhelmed me.

| **The part of this metaphor that stood out to me the most:** |
| --- |
|  |

**Exercise 8.3**

Now that you have dedicated time and effort to constructively confront the sin you wrote about in Exercise 5.1, it is time to let go of any negative thoughts or feelings you may still be experiencing about yourself because of this sin. It’s time to exit the dark room.

Using the rows provided below, make a list of any negative thoughts or feelings you may still be experiencing because of your sin. These might include things like shame, guilt, discomfort, self-doubt, etc. You can add more rows to the table if you need more space.

| **Negative thoughts or feelings I am still experiencing because of this sin:** |
| --- |
| 1. |
| 2. |
| 3. |
| 4. |
| 5. |

**Exercise 8.4**

In Hebrews 12:1, the author tells us to “lay aside every weight and the sin that clings so closely” so that we can run the race marked out for our lives. After we have turned to God and confronted our sin, whatever negative thoughts or feelings we may still be experiencing about ourselves because of our sin are adding to the weight that hinders our ability to run our race. If a heavy backpack makes it more difficult for someone to run, how do you think the negative thoughts or feelings you listed in Exercise 8.3 are affecting your ability to run the race God has prepared for you?

Jesus said, “Take my yoke upon you, and learn from me, for I am gentle and humble in heart, and you will find rest for your souls” (Matthew 11:29). In this exercise, we are going to approach Jesus with the burden of our negative thoughts or feelings and exchange those for the peace he offers. Once you have read through the following instructions, close your eyes to carry out the exercise. Set a timer for five minutes so you can feel comfortable settling into it.

Start with a few deep breaths to settle your mind and calm your body. Find a comfortable sitting position. Put both of your hands on your knees with your palms facing upward. Imagine that Jesus is there with you sitting in the same position directly across from you. You are eye level with one another. His hands are also on his knees with his palms facing upward.

Imagine one of the thoughts or emotions that you listed in Exercise 8.3 is in the palm of your hand. Take note of its size, shape, or color. When you are ready, picture yourself slowly making a fist around this thought or emotion. Then lift up your hand, turn it over, and place it onto the palm of Jesus’ hand. On an out-breath, open your hand so that the thought or emotion has now shifted from your hand to Jesus’ hand. Then imagine Jesus making a fist with his other hand, lifting up that hand, turning it over, and placing it onto the palm of your hand. He opens his hand and says, “my peace I give to you” (John 14:27). Repeat this exercise for each of the thoughts or feelings you listed in Exercise 8.3.

****You may now begin the exercise****

What did you experience as you completed this exercise? Are there any specific words or phrases that capture what you experienced as you swapped your thoughts or feelings with Jesus’ peace? You can add more rows to the table if you need more space.

| **My experience completing this exercise:** |
| --- |
|  |

This exercise was intended for you to experience Jesus’ peace taking root in your heart. Over time, this peace will increase and any lingering negative thoughts or feelings over the sin you have been addressing in this workbook will decrease. But it’s important to understand what this really means.

Sometimes, we think of peace itself in neutral terms, such as when we think of it as an absence of conflict.

But Jesus’ peace is much more than this. It is not merely an absence. It is a *presence*: a presence of God’s perfect love that satisfies our most fundamental need as humans. The apostle Paul experienced this peace himself while imprisoned in Philippi, and during that time he wrote:

^4^Rejoice in the Lord always; again I will say, Rejoice. ^5^Let your gentleness be known to everyone. The Lord is near. ^6^Do not be anxious about anything, but in everything by prayer and supplication with thanksgiving let your requests be made known to God. ^7^And the peace of God, which surpasses all understanding, will guard your hearts and your minds in Christ Jesus (Philippians 4:4-7).

Let Paul’s words dwell in your mind as you rest in the peace found in Jesus.

**Lesson 9 – I am forgiven**

Even after we’ve attempted to make amends for sin, many of us continue to struggle to accept God’s forgiveness. Our reasons for this are many and varied, but at bottom, there is a question that is worthy of serious reflection: “*Which is stronger: sin or the gracious forgiveness of God?”*

One Christian who wrestled seriously with the answer to this question was John Newton (1725-1807). When he was younger, Newton had been captain of a ship involved in the African slave trade. The number of passengers he transported into a life of slavery was immense, and the conditions of the quarters in which they were transported were unspeakably bad. So bad, in fact, that many of them perished on the journey.

In reflecting on this part of his life in retrospect, Newton remained wholly appalled and aggrieved for the part he had played in the slave trade. He wrote about this, saying, “I hope it will always be a subject of humiliating reflection to me, that I was, once, an active instrument, in a business at which my heart now shudders”.^[[4]](#footnote-5)^

Newton wrote these words and the book in which they appear after becoming both a Christian and an Anglican priest. In his years pursuing ordination, Newton developed a friendship with William Wilberforce, a famed abolitionist, who worked alongside him in an effort to alter public perception of the slave trade in Great Britain. But even in attempting to make amends for his part in the slave trade, Newton knew that he could not truly pay back all the harm that he had brought about. It was simply too great. But it was out of his personal anguish that he wrote (probably) the most recognizable hymn in the English language to answer the question of whether grace is stronger than sin, “Amazing Grace”.

**Exercise 9.1**

Let’s listen to “Amazing Grace”. After reading through these instructions, listen to the entire song with your eyes closed. We have provided a weblink to a YouTube version of the song: <https://www.youtube.com/watch?v=EHXTqVmC3JQ>. Click or copy the link into your web browser to listen to this version, or if you have a preferred version, you are welcome to listen to that instead.

****You may listen to the song****

What thoughts, feelings, or physical sensations did you experience as you listened to “Amazing Grace”? Why do you think the song affected you in this way?

| **What I experienced when I listened to the song:** |
| --- |
|  |

The lyrics of “Amazing Grace” are presented below. As you reflect on your experience listening to the song and the lyrics, use the box underneath the lyrics to write the line you appreciate most and explain why that line resonates most strongly with you?

**Amazing Grace**

Amazing grace! How sweet the sound,
  That saved a wretch; like me!
I once was lost, but now am found,
  Was blind, but now I see.

’Twas grace that taught my heart to fear,
  And grace my fears relieved;
How precious did that grace appear
  The hour I first believed!

The Lord hath promised good to me,
  His word my hope secures;
He will my shield and portion be
  As long as life endures.

When we’ve been there ten thousand years,
  Bright shining as the sun,
We’ve no less days to sing God’s praise
  Than when we first begun.

| **The line from “Amazing Grace” that I appreciate most and why:** |
| --- |
|  |

**Exercise 9.2**

In this exercise, we are going to begin by briefly reflecting on a section of Psalm 103. Read the following verses:

^8^ The Lord is merciful and gracious,

slow to anger and abounding in steadfast love.
^9^ He will not always accuse,
 nor will he keep his anger forever.
^10^ He does not deal with us according to our sins
 nor repay us according to our iniquities.
^11^ For as the heavens are high above the earth,
 so great is his steadfast love toward those who fear him;
^12^ as far as the east is from the west,
 so far he removes our transgressions from us.
^13^ As a father has compassion for his children,
 so the Lord has compassion for those who fear him.
^14^ For he knows how we were made;
 he remembers that we are dust.

When God forgives our sin, that sin is so far removed from us that it is no longer in sight when he looks upon us. God no longer holds our sin against us or plans to repay us according to it. For God does not hold grudges; he extends grace.

Another way to put this is to think more deeply about the distance between East and West. If East and West were *destinations*, then the distance between them would be finite and measurable. But they are not destinations; they are *directions*. And they are not only directions, but opposed directions. The idea, then, is this: travel east as far as you wish, even an infinite distance. Never at any point while traveling east will you be in a westward direction. To turn westward, you would have to stop walking east, turn around, and walk west.

**Give it a try.** Right now, where you are, figure out which direction is east and travel a bit in that direction. Now turn around and travel west (just a few feet). Now try to travel both east and west at the same time.

It might seem silly to try and walk both east and west, but experiencing that silliness is part of the point. It’s something we just cannot do. If we apply this idea to God’s forgiveness of us, once God has forgiven our sin, that sin never again registers on God’s radar. So, the psalmist uses this metaphor to help us see how firmly committed God is to only one direction for those who are repentant: namely, the direction of grace.

**Exercise 9.3**

Children love pretend play, especially when dressing up as a favorite character (e.g., superheroes, Disney figures). In these moments, they “put on” the character, sometimes even insisting on being called by their new name.

Think back to your own childhood—was there a character you loved pretending to be? What drew you to them? Perhaps their bravery or a special skill inspired you.

This idea of “putting on” another’s qualities can help us understand God’s forgiveness. When he forgives us, he “covers” us with Christ’s righteousness, seeing us as He sees Jesus—fully righteous. The Apostle Paul describes this transformation:

^1^ There is therefore now no condemnation for those who are in Christ Jesus. ^2^ For the law of the Spirit of life in Christ Jesus has set you free from the law of sin and of death (Romans 8:1-2).

I have been crucified with Christ; and it is no longer I who live, but it is Christ who lives in me. And the life I now live in the flesh I live by the faith of the Son of God, who loved me and gave himself for me (Galatians 2:19b-20).

Do you not know that wrongdoers will not inherit the kingdom of God?**...** But you were washed, you were sanctified, you were justified in the name of the Lord Jesus and in the Spirit of our God (1 Corinthians 6:9 & 11).

If God sees us exactly as he sees Jesus, what qualities do you think God sees? List as many of the qualities of Jesus that God sees in you.

| **Qualities of Jesus that God sees in me:** |
| --- |
|  |

Standing amidst God’s forgiving light, Paul does *not* say you are merely declared righteous. He does *not* say God pretends as if you were righteous. No, what Paul says very explicitly is this: you are *made* righteous in Christ Jesus. Here it is in Romans 5:19 (italics added for emphasis):

For just as through the one man’s [Adam’s] disobedience the many were made sinners, so through the one man’s [Jesus’] obedience the many will be *made righteous*.

Our righteousness is not pretend. It is reality. You are forgiven, and God sees you with those same qualities you listed in the above box for Jesus. So, who are you in Christ Jesus? Write your response in the box below.

| **Who I am in Christ Jesus:** |
| --- |
|  |

**Exercise 9.5**

When Paul says we were made righteous in Christ Jesus (Romans 5:19), he is also making another important point: we are righteous through the work of Jesus on the cross. It’s a *gift* that conquered sin and darkness, and it has been *freely* given to us. After taking a moment to reflect on this, we invite you to say a prayer of gratitude to God for giving you Jesus’ righteousness as a gift.

****You may begin your prayer****

**Venture toward renewal and virtue**

**Lesson 10 – Cultivating habits of holiness**

In Lesson 3, we used the water faucet metaphor to explore how the outpouring of God’s love never ceases. In fact, God’s love for us is so abundant that it doesn’t just fill our cups. It *overflows* them. But how much love we catch and how deep a friendship we cultivate depends (in part) on us, on how much capacity our cup has to receive God’s love. While our sin momentarily diminishes the capacity of our cup to receive the water that represents God’s endless supply of love, our cup may also grow, meaning that our capacity to receive God’s love may increase. This is where the idea of sanctification comes in. Sanctification simply refers to the process of becoming holy or being made sacred by God.

Different parts of scripture address the concept of sanctification. One important place where this concept appears is in Hebrews 10:14, which states:

“by a single offering [Jesus] has perfected for all time those who are sanctified”.

Although this NRSVue translation makes it sound as if the process of our sanctification is complete, the original Greek translated as “are sanctified” here actually implies that the process of our sanctification is ongoing. Crucially, our *justification* is complete (“perfected”), meaning that we are indeed members of God’s body, the church. But this status, this justification, is different from our ongoing sanctification. And to bring this out, a more suitable translation of Hebrews 10:14 would read:

“by a single offering [Jesus] has perfected for all time those who are *being* sanctified”.

Don’t miss this point. Scripture is clear that Jesus’ work of forgiving sin has *perfected* us for all time. That forgiveness is complete, and we can rest in it. We are *clothed* in it. We are wearing the righteousness of the children of God. But even though God’s forgiveness is assured, there is still work to be done: *sanctificatory* work. It is our work with God, cooperatively, to increase further (by his grace) our capacity to receive his love as he shapes our characters after Christ’s likeness. It is this notion of character formation, empowered by the Holy Spirit, that we’ll focus on for this lesson.

**Exercise 10.1**

- 1. **Choose at least one part of your body—for example: your wrists, shoulders, hamstrings, or neck—to stretch out (carefully)**. Remember when you stretch to *breathe* through the stretch, *close your eyes*, and *focus* on that stretching feeling.
  2. **Carefully, spend 30 seconds stretching that part of your body.**
  3. **Read this:** What you experienced while performing that stretch is the limit, the full *capacity*, of your chosen muscle to stretch. You won’t notice tomorrow, but over time, that capacity to stretch (if you continue to work at it) will increase. The same is true of our characters. Working on our flexibility and working on our characters are ongoing, slow processes. But persistence allows us to reap the benefits of both activities.
  4. **Repeat this activity with other parts of your body as needed.** While you continue to stretch, reflect on the comparison just made between stretching and character formation.

Sanctification largely occurs through growing in virtue, which can be defined as an excellence of character (see 2 Peter 1:3-5). This is closely tied to pursuing holiness (see 1 Peter 1:15-16), as scripture tells us we are created to be holy and to develop a character in imitation of Christ. But to do this well, to grow in virtue, requires training, a training that moves us closer to becoming the person, the self, God created us to be—that is, our true self that is most fully capable of receiving God’s boundless love.

There are many virtues, including (but not limited to) traits like courage, temperance, honesty, generosity, faith, hope, and love. However, *true* virtue requires acting with the right motives, not just performing good deeds. For instance, giving to the poor only to improve one’s image isn’t generosity—it’s just performing a good action with a bad motive. As a result, to form virtue, we have to ask ourselves not only the question, “What should I do?” but also, “What should be my reason for doing it?”

Because virtues are habits, they require time to develop, just as mastering an instrument or becoming more flexible do. But even though growing in virtue is a gradual process, by consistently aiming to strengthen our character we can expect to experience incremental, bit-by-bit growth that promises to blossom over time. Learning from our mistakes is key to our growth. And keep in mind: each day offers us opportunities to progress toward the virtuous self we aspire to be. Taking advantage of such opportunities will yield, by God’s grace, real change for the better in the long run.

**Exercise 10.2**

We develop virtue through daily habits (not major leaps) in relationship with God. There are many ways we can do this. In this workbook, we invite you to try and commit to a specific daily habit of praying the Examen. The Examen is a style of prayer that comes to us from Saint Ignatius, who claimed that “God is in all things.” As such, he had great faith that we could feel the stirrings of God inside us in many kinds of moments.

There are some moments when we feel at peace or a kind of satisfied joy. Ignatius called these *consolations*, times when God feels near to us. In other moments, we feel out of sorts, anxious, or restless. These he called *desolations*, times when God feels more distant from us. The Examen is a prayer that can help us to reflect on our day and identify those moments of consolation and desolation.

The times that lead to consolation and desolation may surprise you. They may occur when you are alone or when you are with others. When you reflect on your experiences of consolation or desolation, you might realize that certain people or activities play a part in helping you to experience greater closeness with (or distance from) God. By reflecting on such experiences each day, we can notice more of God’s presence in different moments of our life. This helps us form the habit of noticing God, being aware of his presence. If we pay attention to those activities and moments that lead to consolation, the Examen will provide indications of what activities and choices might best lead us to deeper friendship with God.

Use the guidelines that follow to complete the Examen for today.

**The Examen**

**Step 1: Make yourself aware of God**. God is present with you (sustaining your existence at every moment). Quiet your body and mind, and ask God to reveal his presence to you.

**Step 2: Review your day as if it were a movie playing before your eyes**. After you have gone through the day, identify at least 3 moments that you are grateful for. Thank God for those moments.

**Step 3: Think about the emotions you experienced today.** When did you feel at peace or joyous today? *These are your consolations.* Using the **green** boxes below, briefly describe which activities brought you consolation and why? There are three green rows provided, but you can add more rows if you have more than three activities to list. We have provided some examples to help guide you (and feel free to make these your own).

| **Activities that brought me consolation (feelings of peace) today:** |
| --- |
| *Example 1*: Walk along the river. |
| *Example 2*: Sitting in silence with a cup of tea to begin my day in prayer. |
| 1. |
| 2. |
| 3. |

**Step 4: Think once more about the emotions you experienced today.** When did you feel uneasy, anxious, or angry? *These are your desolations.* Using the **orange** boxes below, briefly describe which activities caused you desolation and why? There are three orange rows provided, but you can add more rows if you have more than three activities to list. We have provided some examples to help guide you.

| **Activities that caused me desolation (spiritual unrest) today:** |
| --- |
| *Example 1*: Being honked at by another driver. |
| *Example 2*: Being misunderstood by my colleague at work. |
| 1. |
| 2. |
| 3. |

**Step 5: Think about one moment of desolation or consolation that you would like to bring to God.** Reflect on that moment. What do you want to say to God about that part of your day? Say it to him now.

**Step 6: Tell God what you are looking forward to about tomorrow**. Tomorrow is a new opportunity to grow, learn, and love.

**Exercise 10.3**

In Exercise 10.2, you completed the Examen for one specific day following a six-step process. Let’s consider this day 1. We now invite you to go through the Examen at the end of each day (for example, before going to sleep) for at least 3 more days. We encourage you to set time aside to go through the Examen at least 4 times in total before moving onto Exercise 10.4.

At the end of your day for the next 3 days, pray the Examen following the six steps provided in Exercise 10.2. When you get to steps 3 and 4, use the boxes provided below to describe the activities that brought you consolation and desolation on each day.

**Day 2**

Write the date you are going through the Examen for the second time in this workbook: [dd/mm/yyyy]

Using the **green** boxes below, briefly describe which activities brought you consolation today and why? There are three green rows provided, but you can add more rows if you have more than three activities to list.

| **Activities that brought me consolation today (day 2):** |
| --- |
| 1. |
| 2. |
| 3. |

Using the **orange** boxes below, briefly describe which activities caused you desolation today and why. There are three orange rows provided, but you can add more rows if you have more than three activities to list.

| **Activities that caused me desolation today (day 2):** |
| --- |
| 1. |
| 2. |
| 3. |

**Day 3**

Write the date you are going through the Examen for the third time in this workbook: [dd/mm/yyyy]

Using the **green** boxes below, briefly describe which activities brought you consolation today and why. There are three green rows provided, but you can add more rows if you have more than three activities to list.

| **Activities that brought me consolation today (day 3):** |
| --- |
| 1. |
| 2. |
| 3. |

Using the **orange** boxes below, briefly describe which activities caused you desolation today and why. There are three orange rows provided, but you can add more rows if you have more than three activities to list.

| **Activities that caused me desolation today (day 3):** |
| --- |
| 1. |
| 2. |
| 3. |

**Day 4**

Write the date you are going through the Examen for the fourth time in this workbook: [dd/mm/yyyy]

Using the **green** boxes below, briefly describe which activities brought you consolation today and why. There are three green rows provided, but you can add more rows if you have more than three activities to list.

| **Activities that brought me consolation today (day 4):** |
| --- |
| 1. |
| 2. |
| 3. |

Using the **orange** boxes below, briefly describe which activities caused you desolation today and why. There are three orange rows provided, but you can add more rows if you have more than three activities to list.

| **Activities that caused me desolation today (day 4):** |
| --- |
| 1. |
| 2. |
| 3. |

**Exercise 10.4**

In Exercise 10.3, you listed the activities during which you experienced consolations and desolations in each of 4 days. In this exercise, you are going to review those activities to identify any patterns of consolation and desolation that occurred across those 4 days.

Using the **green** boxes below, list the activities in which you experienced consolation the ***most***. We have provided some examples as a guide. There are three green rows provided, but you can add more rows if needed.

| **Activities that I experienced the most consolation:** |
| --- |
| *Example 1*: When I was spending time with my friends. |
| *Example 2*: When I prayed. |
| 1. |
| 2. |
| 3. |

Using the **orange** boxes below, list the activities in which you experienced desolation the ***most***. We have provided some examples as a guide. There are three orange rows provided, but you can add more rows if needed.

| **Activities that I experienced the most desolation:** |
| --- |
| *Example 1*: When I was scrolling on social media. |
| *Example 2*: When driving home at the end of the day. |
| 1. |
| 2. |
| 3. |

We are going to draw on these lists to formulate an “if-then” statement designed to help you experience more consolation and less desolation. An “if-then” statement is a goal-setting strategy in which a person identifies a specific situation (“if”) and then defines a specific action they will take in response to that situation (“then”).

To begin, choose one of the three desolation activities from the list that you would like to focus on. This is the specific situation that will form the “if” part of your “if-then” statement. The “then” part of the statement needs to be a specific action you will take when you are confronted with the specific “if” situation. For this, you might choose one of the three consolation activities you listed above. Drawing on the examples we provided, if someone experiences desolation when scrolling on social media and consolation when they pray, a suitable “if-then” statement might be:

*IF I notice that I am spending unnecessary time on my phone scrolling through social media, THEN I will put aside my phone and pray.*

Following this example, use the box below to formulate an “if-then” statement using the activity of desolation you chose and an activity of consolation that is a suitable replacement. Make sure that this “if-then” statement is something you are willing to commit to doing.

| **My “if-then” statement to replace desolation with consolation:** |
| --- |
| If I [INSERT ACTIVITY OF DESOLATION], then I will [INSERT ACTIVITY OF CONSOLATION]. |

The Examen prayer helps us to encounter God in daily life by growing in awareness of the Holy Spirit moving in us when we experience consolation and desolation. It is very powerful to become aware of God’s presence in daily life. Try to practice this awareness by recognizing when you are engaged in your chosen activity of desolation. When you do, try to apply your “if-then” statement to replace your activity of desolation with one of consolation. If you are able to do this consistently, you may find yourself developing a new habit that draws you nearer to God’s loving presence.

**Exercise 10.5**

The author of Hebrews 10:25 encourages Christians to not neglect meeting together, for in community and shoulder-to-shoulder we are far better at resisting sin and pursuing the goodness God desires for us.

Empirical studies have borne out the truth of this biblical wisdom. Human beings all tend to succeed more in achieving their goals—whether those goals are to become a better writer or to address some societal need—when they pursue those goals with others. Below is a list of ways you can pursue virtue in community.

After you have taken some time to reflect and pray about this list, we encourage you to choose at least one of the activities and commit to doing it.

1. Attend Church Service
2. Join a Small Group at a Church
3. Engage in Local Community Service
   1. Volunteer at a Food Pantry
   2. Volunteer at a Homeless Shelter
   3. Volunteer at a Community Event
4. Join an Online Prayer Group
5. Commit to being more consistent in something you are already doing along the lines of the activities listed above

Which of the activities listed above are you making a commitment to doing (or doing more consistently)? Use the box provided below to name the specific activity (or activities).

| **The communal activity that I have committed to doing (or doing more consistently):** |
| --- |
|  |

To further solidify your commitment to the specific activity or activities, write the name of one trusted friend you will tell about this commitment.

| **The friend I will tell about this commitment is:** |
| --- |
|  |

**Exercise 10.6**

In this lesson, we have seen that growing in character is part of our ongoing work of sanctification and that such work is empowered by the Holy Spirit. In addition, we have practiced the Examen to listen better to God’s direction as he guides us to experience deeper friendship (consolation) with him, and we have considered how God might help us through our reliance on different communities of faith in which we can be encouraged in our journey to virtuous living.

We now invite you to sign your name to the following commitment. In it, you commit again to live in a way that reflects God’s values, especially during trials, temptations, or times of desolation.

**My Personal Commitment to God**

**I, [INSERT YOUR FULL NAME] declare to myself that as of the date [INSERT MONTH AND DAY], [INSERT YEAR], I commit to living in accordance with God’s values and according to his will. I make this commitment while knowing that God walks alongside me as I seek to be more like Jesus. I am not alone now, nor will I ever be alone, for God is with me. I will venture forth toward virtue, and though I acknowledge that I may stumble, I promise to nevertheless press on in this commitment, for I can see clearly in the light of God’s love for me the path to righteousness has been paved.**

**Establish a plan to keep experiencing God’s forgiveness**

**Lesson 11 – What can I do when I doubt God’s forgiveness?**

At different points throughout this workbook, we have considered how God’s love for us involves his deep desires for our good and for friendship with us. We have also reflected on the fact that God’s love for us never ceases, even if our sins might momentarily reduce our capacity to experience God’s love. But we know, too, that we are forgiven. And when we experience that forgiveness, our capacity for receiving divine love has been restored. In many ways, though, that capacity is not simply restored to what it was before the sin. It may very well be *greater* than it was.

Consider the Japanese art of repairing broken pottery known as *kintsugi*. Those who practice this art take broken shards of pottery and repair them by joining together the cracks with golden lacquer. The resulting pottery does not hide the cracks but, instead, emphasizes the repair done to them in a way that makes the previously disjoined parts more valuable and beautiful than the original, unbroken item (see below).


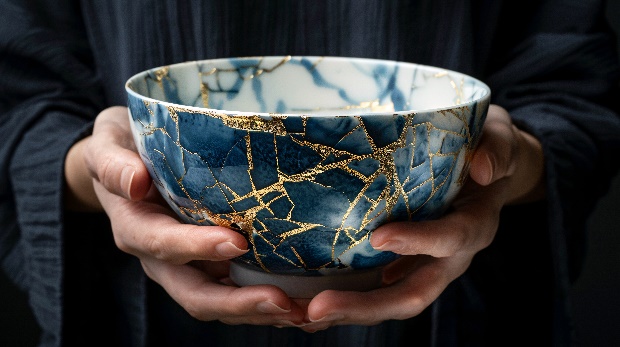


Something similar can be said of our sin. Although no one wants to be stuck with the results of sin (any more than someone wants to be stuck with broken pottery shards), it is a mistake to think that once sin occurs we and God have both been defeated. For out of the broken shards of our mistakes, out of the weakness of our human condition, God can bring triumph. His forgiveness repairs our weakened capacity to hold his love for us and that very act of forgiveness serves as an eternal testament to God’s love for us as individuals.

We find examples of this throughout scripture. Jesus’ death was in some sense an event that God didn’t want to transpire, but once the death occurred, God responded with resurrection, marking the most incredible event since the beginning of creation. He took that which was broken and out of it made something beautiful, even priceless.

God sees every Christian’s story this way. Sin brings all kinds of things we don’t want into our lives and the lives of others. But God, like the *kintsugi* master, knows exactly how to repair what’s gone wrong. And in doing this, God’s forgiveness marks the value of your deep friendship to him (like a golden lacquer), which brings us out of our sin into more abundant life, a life full of gratitude and courage.

**Exercise 11.1**

Even when we fully know and accept God’s forgiveness, it is not uncommon for people to experience moments when they have doubts over whether God has forgiven them for their past sin. Or they may encounter some of the negative thoughts or feelings (e.g., guilt, shame) that were directed to themselves around the time they were first struggling with a particular sin. If we encounter these moments after we have confronted our sin and fully addressed it with God, we need to be prepared to respond effectively.

Peter—the apostle to whom Jesus said, “you are Peter, on this rock I will build my church, and the gates of Hades will not prevail against it” (Matthew 16:18)—is remembered as an apostle characterized by boldness and devotion of the highest order to God. But Peter gravely sinned when he abandoned Jesus and denied any association with him, even as he hung on the cross.

In the gospels, Peter seems to struggle with forgiving himself for this sin, but because we have this story, we have something we can look to as a guide for how to hold onto divine forgiveness. You see, Peter denied Christ *three* times, but when we turn to the end of the gospel of John, he encounters the resurrected Jesus again who knows fully what Peter has done. In that knowledge, though, Jesus reaffirms his relationship to Peter *three* times, reminding Peter that no matter how many times he has sinned, Jesus’ forgiveness will be there for him.

Like us, Peter struggled to hold onto forgiveness because he simply would not forget what he had done to Jesus. He responded in this moment, though, by turning to the very Word of God. We too, then, can respond in these moments ourselves by turning to the word, reciting scripture that we have memorized.

1. **Choose a scripture verse that emphasizes God’s love for you and victory over sin. Here are some possibilities:**

- Romans 8:1: “Therefore there is now no condemnation for those who are in Christ Jesus”.
- 1 John 3:1a: “See what love the Father has given us, that we should be called children of God, and that is what we are”.
- Galatians 2:19b-20: “I have been crucified with Christ, ^20^ and it is no longer I who live, but it is Christ who lives in me. And the life I now live in the flesh I live by the faith of the Son of God, who loved me and gave himself for me”.

1. **Write the verse on a small piece of paper and tape the verse above your bathroom sink.**
2. **Every time you brush your teeth, take the time to speak the verse out loud before brushing.**

Before you know it, you will have memorized this verse. Whenever you feel difficulty believing that God has forgiven you, bring that verse before your mind again. Feel free to place similar passages in different locations around your home that you visit frequently.

**Exercise 11.2**

Memorizing scripture is just one way we can prepare ourselves to effectively respond to moments when we face doubts over God’s forgiveness of our past sin. In the table below, we have included two categories of supports—self and other people—that could help in these instances. As a matter of principle, God is at work in you and in others all the time, so God’s presence is implicit as a support through all of these activities or individuals.

Under each category, we have listed examples of what those supports might look like. This is not a complete list of applicable supports within each category, so feel free to add more rows and indicate any additional supports that come to mind.

After you have added any other supports to the table, choose two supports from *each* column that you could plan to draw on during moments of doubt over whether God has forgiven you for the specific sin you wrote about in Lesson 5. Use **bold** or a **color** to mark your selection from each column.

| **Self** | **Other people** |
| --- | --- |
| 1. Recite verses from Exercise 11.1 | 1. Talk to a spiritual leader |
| 2. Read Psalm 23 | 2. Talk to an accountability partner |
| 3. Read through this workbook | 3. Share my doubts with the small group I am part of |
| 4. Sing along with a favorite hymn or other sacred song | 4. Talk with a family member |
| 5. Pray | 5. Pray |
| 6. | 6. |
| 7. | 7. |
| 8. | 8. |

Now that you have identified some supports to draw on during moments you experience doubt over God’s forgiveness of the specific sin you addressed in this workbook, *write a plan* of how you intend to draw on these supports if/when future doubts arise. If any of the supports that you have selected require you to take some preliminary steps to put them in place, *describe how you will do this*. For example, if you selected “talk to an accountability partner” but you don’t already have a Christian who is supporting you in this way, then describe how you will go about finding a suitable person.

| **My plan if I have doubts over God’s forgiveness for this specific sin in the future:** |
| --- |
|  |

**Exercise 11.3**

In the early 16^th^ century, Matthias Grünewald painted *Crucifixion* (see below). This painting is well-known for its emphasis on the extent of Christ’s suffering, displaying the depths to which he was lovingly prepared to go for the forgiveness of our sins.


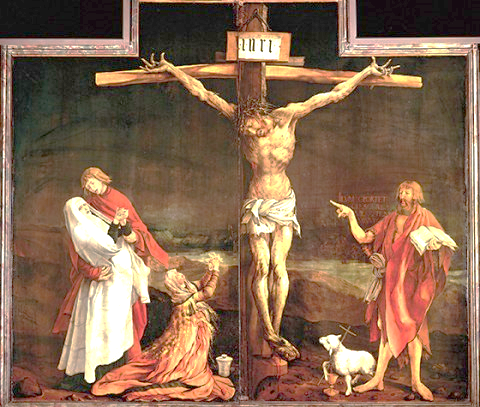


Grünewald painted this for display at St. Anthony’s monastery hospital, which was home to people with a particular skin disease (ergotism). In the painting, Jesus bears similar skin afflictions to those people who would have lived at St. Anthony’s (you may be able to see some of those skin lesions on Jesus’ chest and legs). Grünewald’s painting served to remind those at the hospital that Christ entered the crucifixion so that we might not bear our afflictions alone. He knows what we have suffered, he knows what wrongs we’ve done, but those things give him no reason whatsoever to abandon us. Instead, his deep knowledge of where we’ve been and who we are inspires nothing short of the greatest act of love.

As you reflect on your journey through this workbook, which lesson or exercise helped you to experience more of God’s loving presence? Why do you think this is the case?

| **A lesson or exercise that helped me to experience more of God’s loving presence:** |
| --- |
|  |

The lesson or exercise you described in the box above is one example of how God willingly enters into our struggles with sin and bears the burden of those struggles alongside us. If God has journeyed with you through this workbook, imagine how eager he is to journey with you throughout your life as you move forward. We hope that the moments you experienced God’s loving presence during this workbook will serve as reference points that you will draw on when you face doubts about God’s forgiveness or struggles over sin.

**Exercise 11.4**

The fact that Christians now hold up the cross as a symbol of faith would’ve been shocking to the Romans of Jesus’ day. It was literally an instrument of execution, and as such, is not something one normally wears as a necklace or bracelet.

Given the strangeness of the cross as a symbol of Christianity, it is helpful to contrast Grünewald’s painting with icons of the crucifixion found in the Eastern Orthodox tradition of Christianity (see below).


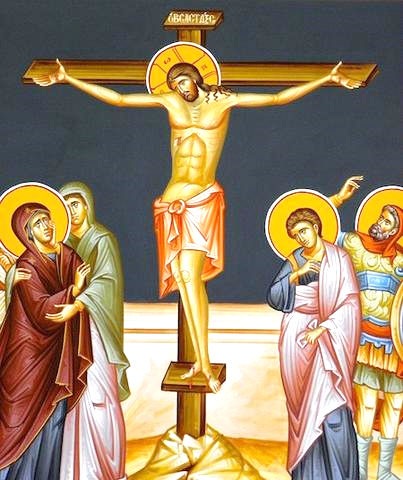


The Orthodox do not depict Jesus as suffering greatly on the cross. Notice, for instance, how in this icon Jesus almost appears to be holding himself up on his own two feet. This artistic choice does not use suffering to make its point. Rather, it emphasizes that God and Jesus were fully in control of what was happening at the cross; it was an absolute *victory* over sin and death. And it is the victory, rather than the struggle, that the Orthodox want us to see whenever we approach the cross of Christ. It is a symbol of Christianity because instead of a symbol of death it is now a symbol of life for all of us who are in Christ Jesus.

You may not have thought about it this way, but the time and effort you have invested in this workbook is part of Jesus’ ultimate victory of sin and death because you are not letting sin have the last word. That role is instead filled by God’s love, and by accepting God’s loving forgiveness (or making way for that acceptance) we invite God’s love to transform us and be victorious.

As you reflect on your experience completing this workbook, what do you think makes your journey through this workbook a victory? As you write, you may find it helpful to consider some of the following:

- How has your understanding of God or your relationship with God changed since before you completed this workbook?
- After which lesson(s), if any, did you feel closest to God? Why do you think this was the case?
- How has the process of completing this workbook changed you?

| **How my experience completing this workbook is a victory:** |
| --- |
| My experience completing this workbook is a victory because… |

What we have considered in this lesson are several different ways to frame redemption. One metaphor illustrates how, even when something has gone wrong, when a bowl has shattered, the hands of a *kintsugi* master can create something even more beautiful out of the broken shards. We also saw how the apostle Peter experienced similar repair in his own life when Jesus affirmed his love for Peter over and over again. And, lastly, we looked at the emphasis of victory found in icons of the crucifixion from the Orthodox tradition of Christianity. Even in the face of the end of life itself, God brings new, everlasting creation.

So, go now. Rest in God’s love for you. For when God thinks of you (and he always does) he thinks of you as he thinks of Christ: beloved and of immeasurable value.

1. See Bernard of Clairvaux, *On Loving God. Translated by Paul Halsall* (Christian Classics Ethereal Library): <https://ccel.org/ccel/bernard/loving_god//loving_god> [↑](#footnote-ref-2)
2. Reinhold Niebuhr, *The Nature and Destiny of Man*, 185. [↑](#footnote-ref-3)
3. Augustine, *The Confessions,* book 1, chapter 1. This can be accessed through the Christian Classics Ethereal Library at <https://www.ccel.org/ccel/schaff/npnf101.html> [↑](#footnote-ref-4)
4. John Newton, *Thoughts Upon the African Slave Trade.* This quotation comes from the beginning of that book where Newton sets down his reasons for writing it. [↑](#footnote-ref-5)
